# Comparison of Clinical Characteristics and Treatment Outcomes of Children Selected for Treatment of Severe Acute Malnutrition Using Mid Upper Arm Circumference and/or Weight-for-Height Z-Score

**DOI:** 10.1371/journal.pone.0137606

**Published:** 2015-09-16

**Authors:** Sheila Isanaka, Benjamin Guesdon, Amy S. Labar, Kerstin Hanson, Celine Langendorf, Rebecca F. Grais

**Affiliations:** 1 Department of Research, Epicentre, Paris, France; 2 Department of Nutrition, Harvard T.H. Chan School of Public Health, Boston, United States of America; 3 Department of Global Health and Population, Harvard T.H. Chan School of Public Health, Boston, United States of America; 4 Action Contre la Faim, Paris, France; 5 Médecins Sans Frontières, Paris, France; Instituto de Ciências Biomédicas / Universidade de São Paulo—USP, BRAZIL

## Abstract

**Objectives:**

Debate for a greater role of mid-upper arm circumference (MUAC) measures in nutritional programming continues, but a shift from therapeutic feeding programs admitting children using MUAC and/or weight-for-height Z (WHZ) to a new model admitting children using MUAC only remains complicated by limited information regarding the clinical profile and response to treatment of children selected by MUAC vs. WHZ. To broaden our understanding of how children identified for therapeutic feeding by MUAC and/or WHZ may differ, we aimed to investigate differences between children identified for therapeutic feeding by MUAC and/or WHZ in terms of demographic, anthropometric, clinical, and laboratory and treatment response characteristics.

**Methods:**

Using secondary data from a randomized trial in rural Niger among children with uncomplicated severe acute malnutrition, we compared children that would be admitted to a therapeutic feeding program that used a single anthropometric criterion of MUAC< 115 mm vs. children that are admitted under current admission criteria (WHZ< -3 and/or MUAC< 115 mm) but would be excluded from a program that used a single MUAC< 115 mm admission criterion. We assessed differences between groups using multivariate regression, employing linear regression for continuous outcomes and log-binomial regression for dichotomous outcomes.

**Results:**

We found no difference in terms of clinical and laboratory characteristics and discharge outcomes evaluated between children that would be included in a MUAC< 115 mm therapeutic feeding program vs. children that are currently eligible for therapeutic feeding but would be excluded from a MUAC-only program.

**Conclusions:**

A single anthropometric admission criterion of MUAC < 115 mm did not differentiate well between children in terms of clinical or laboratory measures or program outcomes in this context. If nutritional programming is to use a single MUAC-based criterion for admission to treatment, further research and program experience can help to identify the most appropriate criterion in a broad range of contexts to target children in most urgent need of treatment.

## Introduction

It is estimated that 34 million children under the age of 5 each year are affected by severe acute malnutrition (SAM), a condition associated with significant increased risks of mortality and morbidity [1, 2]. In 2007, a joint United Nations statement endorsed a new model for the management of SAM that combines outpatient treatment with ready-to-use therapeutic foods (RUTF) for uncomplicated cases and inpatient treatment for complicated cases [3]. This model has been shown to be both effective [4, 5] and cost-effective [6–8], with the potential to bring life-saving treatment to millions of children.

Since 2009, the World Health Organization (WHO) has recommended using weight-for-height Z-score (WHZ) < -3 and/or mid-upper arm circumference (MUAC) < 115 mm as anthropometric criteria for admission to therapeutic feeding programs [9, 10]. In recent years, however, the use of MUAC alone for admission has been increasingly discussed [11–13]. Many benefits of using MUAC exist: MUAC is predictive of death, easy to use, acceptable, and linked to community-based screening methods [14, 15]. However, it is known that MUAC and WHZ select different children for treatment [16, 17], complicating a shift from programs currently admitting children using MUAC < 115 mm and/or WHZ < -3 to a new model admitting children using MUAC < 115 mm only. Depending on context, up to 63–79% of children currently recommended for therapeutic feeding with WHZ < -3 and/or MUAC < 115 mm would not be eligible if using MUAC < 115 mm alone for admission [10, 17, 18].

To inform decision making regarding the use of MUAC as a standalone admission criterion in nutrition programming, more information on the programmatic implications of using MUAC alone is urgently needed. Preliminary reports demonstrate demographic and anthropometric differences among children identified by WHZ and MUAC: MUAC is more likely to identify children that are younger, female and more stunted [19–21]. These data have been used to suggest a role for MUAC to identify children that are potentially more vulnerable or at a higher risk of death, supporting the transition to a MUAC-only based admission criterion. Published evidence is, however, scarce and limited in breadth, due to the narrow scope of routine program data often available for analysis. Important parameters, including the clinical profile and treatment response, of children who are currently eligible for therapeutic feeding but would not be recommended for treatment using MUAC < 115 mm as a single anthropometric criterion for treatment remain poorly documented.

To broaden our understanding of how children identified for therapeutic feeding by MUAC and/or WHZ may differ, and to inform future program planning if MUAC-based programming is to occur, we analyzed data from a randomized trial of children aged 6 to 59 months with uncomplicated SAM in Niger. The objective of this analysis was to compare children that would be admitted for outpatient SAM treatment in a MUAC-based program vs. children that are admitted under current admission criteria of low WHZ and/or low MUAC but would be excluded if using MUAC-based admission criteria. We aimed to specifically investigate differences in demographic and anthropometric characteristics between groups previously reported, as well as extend our understanding on other potential differences in vulnerability in terms of clinical and laboratory characteristics and treatment response. These characteristics were also used to explore possible variability within a potential MUAC-only program, comparing children admitted by MUAC < 115 mm admission criteria, with vs. without WHZ < -3. These findings will be useful to describe the potential implications of MUAC-based programming in terms of the clinical needs of the expected patient population and can inform planning if such programs are to be implemented.

## Materials and Methods

### Study Setting

The present study is a secondary analysis of a randomized trial in Niger to assess the impact of systematic antibiotic use in the outpatient treatment of uncomplicated SAM on nutritional recovery (ClinicalTrials.gov number, NCT01613547). The trial protocol was approved by the Comité Consultatif National d’Ethique of the Ministry of Public Health of Niger and the Comité de Protection des Personnes, Ile-de-France and parental informed consent was obtained for all trial participants (S1 Trial Protocol). The study was conducted in Madarounfa, Niger, a rural health district located in the south-central part of the country bordering Nigeria. Niger is one of the poorest and most vulnerable countries in the world, ranking last in 2014 on the Human Development Index with high rates of child mortality and malnutrition [22–24]. Located in the Sahel, household food production is linked to rain-fed agriculture, where staple crops such as millet and sorghum are harvested once per year. Each year, the decrease in food quantity and quality experienced in the months preceding the harvest and the concurrent increase in infectious illness, including diarrhea, pneumonia and malaria, are associated with a seasonal increases in acute malnutrition among children < 5 years of age.

### Definition of Study Population and Procedures

The study population included 2,399 children enrolled in the parent trial from 2012 to 2013. Inclusion criteria for the trial required participants be eligible for outpatient SAM treatment; resident within 15 km of a study health center; available for the 12-week study period; not have been admitted to a nutritional program within the last 3 months; have no clinical complication requiring antibiotic treatment at admission; have not received any antibiotic within the last 7 days; and have no congenital abnormality that may affect growth. A child was considered eligible for outpatient SAM treatment in the trial with 6 and 59 months of age; weight-for-height Z (WHZ) score < -3 according to the 2006 World Health Organization Growth Standards and/or MUAC < 115 mm; sufficient appetite according to a test feeding of RUTF; and absence of clinical complications requiring hospitalization, including bipedal edema. All study children were randomized to amoxicillin (80 mg/kg/day) or placebo for 7 days and received standard nutritional and medical care according to guidelines of the Government of Niger and Médecins Sans Frontières (MSF). At inclusion, children received RUTF (170 kcal/kg/day; Plumpy’nut, Nutriset, France), routine medicines (e.g. folic acid and anti-helminthic treatment, and if appropriate measles vaccination and anti-malarial treatment) to treat and prevent complications and a comprehensive physical exam with systematic assessment of hemoglobin, HIV status and malaria infection by rapid test. Stool, urine and blood samples were collected at admission for bacteriological analysis among a sub-sample of children of approximately 1000 children between October 2012 to October 2013. Weekly follow-up was conducted at the health facility until nutritional recovery or transfer to inpatient care for a maximum of 8 weeks. At each weekly visit, weight to the nearest 100 g; length (children <24 months of age) or standing height (children ≥ 24 months of age) to the nearest 0.1 cm; and MUAC to the nearest 0.1 cm were measured using standard techniques, a physical exam was conducted, and the next weekly ration of RUTF was provided until discharge. Nutritional recovery was defined at or after 3 weeks with WHZ ≥ -2 and MUAC ≥ 115 mm on two consecutive visits; no acute complication or edema for at least 7 days; and completion of all antibiotic and anti-malaria treatment at the time of discharge. Transfer to inpatient care was initiated in case of weight loss > 5% between two consecutive visits or lack of weight gain after 2 weeks and/or clinical complication requiring inpatient management. Non-response was defined as not meeting the criteria for nutritional recovery at 8 weeks.

### Outcome Measures

Demographic characteristics included child age (months) and sex. Weight-for-height (WHZ) and height-for-age (HAZ) were calculated using the 2006 WHO Growth Standards [25], with Z scores < -2 indicative of wasting and stunting, respectively. Clinical characteristics evaluated at admission included fever (axillary temperature ≥ 38.5⁰ C); tachypnea (average of two measures; respiratory rate > 50 breaths per minute for children 6–11 months of age and > 40 breaths per minute for children 12–59 months of age); diarrhea, nasal discharge, cough, and vomiting assessed by clinical examination; anemia (hemoglobin < 11 g/dL; HemoCue Hb 301, HemoCue, Angelholm, Sweden), positive HRP2 rapid diagnostic test for malaria (SD Bioline Malaria Antigen P.f, Standard Diagnostics Inc, Republic of Korea), as well as laboratory-confirmed bacteremia, bacteriuria, and bacterial gastroenteritis (defined as positive stool culture with known pathogen and diarrhea).

In addition, we considered 4 types of program outcomes: recovery, transfer to inpatient care, default, and death. Among all children and those who recovered, we calculated weight gain (g/kg/day) and MUAC gain (mm/day) from admission to week 1 and week 2. Among children who recovered, we calculated length of stay, defined as the number of days between the date of discharge and admission.

### Statistical Analysis

The first comparison presents children that would be included in a MUAC < 115 mm-only therapeutic feeding program (hereafter “MUAC < 115 mm” children) vs. children that are currently eligible for therapeutic feeding using WHZ < -3 and/or MUAC < 115 mm criteria but would be excluded from a MUAC-only program using MUAC < 115 mm for admission (hereafter “MUAC ≥ 115 mm and WHZ < -3” children). The second comparison looks within the hypothetical program population identified by MUAC < 115 mm and compares children with and without WHZ < -3 (hereafter “MUAC < 115 mm and WHZ < -3” and “MUAC < 115 mm and WHZ ≥ -3”, respectively).

Differences between groups adjusting for the parent trial regimen (amoxicillin vs. placebo) were assessed using multivariate regression, employing linear regression for continuous characteristics, ordinal logistic regression for ordinal characteristics, and log-binomial regression for dichotomous. In secondary analyses, we additionally adjusted for age (± 24 months), sex, stunting (HAZ ± -2) at admission. We hypothesized that differences between groups may be limited to certain subgroups and explored possible effect modification by age (± 24 months), sex and stunting (HAZ ± -2). Statistical interactions were therefore assessed using linear regression for continuous outcomes and log-binomial regression for dichotomous outcomes. If a significant interaction was found, the appropriate stratified estimates are presented. Stata 12 (StataCorp, College Station, Texas, USA) was used for statistical analysis. A P-value < 0.05 was considered statistically significant.

## Results

2,399 children aged 6–59 months with MUAC < 115 mm and/or WHZ < -3 were included in the analysis. Overall, children had a median age of 15 months (IQR: 10–22 months), and there was a nearly equal proportion of girls and boys. The distribution of children across anthropometric groups was as follows: 929 (39%) children with MUAC < 115 mm only, 530 (22%) children with WHZ < -3 only, and 940 (39%) children with both MUAC < 115 mm and WHZ < -3. One (<0.1%) child was confirmed to be HIV-positive.

To describe potential differences among children who would be included in a MUAC < 115 mm program vs. those who are currently eligible but who would be excluded using MUAC < 115 mm alone for admission, the first comparison presents children with MUAC < 115 mm (n = 1869) vs. children who would be excluded from a MUAC-based program having MUAC ≥ 115 mm and WHZ < -3(n = 530) (**Table 1**). Children with MUAC < 115 mm were more likely to be younger, female (57% vs. 25%) and stunted (81% vs. 73%), compared to children with MUAC ≥ 115 mm and WHZ < -3. Clinical characteristics on admission and program outcomes did not significantly differ between groups. The prevalence of bacterial infection was low in this population and did not differ between groups. Immediate response to treatment, as evidenced by the rate of weight gain from admission to week 1 and week 2, was greater among children with MUAC ≥ 115 mm and WHZ < -3, compared to MUAC < 115 mm children though ultimately there was no difference in length of stay between groups. Results remained largely consistent with adjustment for age, sex and stunting.

**Table 1:** Demographic and clinical characteristics on admission and program outcomes of children with SAM, by MUAC- and WHZ-defined admission criteria.

Child characteristics	MUAC < 115 mm	MUAC ≥ 115 mm and WHZ < -3	P*	P**
N (%)	1869 (78%)	530 (22%)		
**Demographic characteristics on admission**				
Age, n (%)			< .001	< .001
6–11 months	762 (41%)	91 (17%)		
12–23 months	783 (42%)	275 (52%)		
24–59 months	324 (17%)	164 (31%)		
Females, n (%)	1062 (57%)	134 (25%)	< .001	< .001
**Anthropometric status on admission**				
WHZ, mean (SD)				
Overall	-3.00 (0.69)	-3.37 (0.24)	< .001	< .001
Age < 24 months	-2.95 (0.69)	-3.33 (0.24)	< .001	< .001
Age ≥ 24 months	-3.25 (0.61)	-3.46 (0.23)	< .001	0.003
Boys	-3.25 (0.65)	-3.38 (0.24)	< .001	0.005
Girls	-2.81 (0.66)	-3.34 (0.24)	< .001	< .001
MUAC (mm), mean (SD)				
Overall	111 (37)	118 (24)	< .001	< .001
HAZ < -2	111 (38)	118 (24)	< .001	< .001
HAZ ≥ -2	112 (27)	118 (24)	< .001	< .001
HAZ, mean (SD)	-3.04 (1.22)	-2.81 (1.21)		< .001
HAZ <-2, n (%)				
Overall	1509 (81%)	388 (73%)	< .001	< .001
Age < 24 months	1206 (78%)	239 (65%)	< .001	< .001
Age ≥ 24 months	303 (94%)	149 (91%)	< .001	0.12
**Clinical characteristics on admission**				
Diarrhea	589 (32%)	170 (32%)	0.82	0.90
Nasal discharge	426 (23%)	126 (24%)	0.64	0.63
Cough	308 (16%)	79 (15%)	0.38	0.28
Vomiting	112 (6%)	26 (5%)	0.34	0.36
Tachypnea				
Overall	27 (1%)	4 (1%)	0.23	0.28
Boys	15 (2%)	1 (0.25%)	0.05	0.08
Girls	12 (1%)	3 (2%)	0.47	0.33
Fever [Temperature ≥ 38.5⁰ C]	101 (5%)	31 (6%)	0.70	0.52
Anemia [Hb < 11 g/dL]	1374 (74%)	372 (70%)	0.14	0.28
Malaria positive	1040 (56%)	287 (54%)	0.56	0.18
**Laboratory findings on admission**				
Bacteremia	29 (3%)	12 (5%)	0.23	0.44
Enterobacteriaceae	26 (3%)	8 (3%)	0.78	0.81
Bacteriuria	22 (4%)	4 (2%)	0.39	0.33
Enterobacteriaceae	21 (3%)	3 (2%)	0.24	0.18
Bacterial gastroenteritis	89 (10%)	25 (11%)	0.93	0.98
Enterobacteriaceae	53 (6%)	13 (6%)	0.68	0.52
*Campylobacter spp*.	42 (5%)	13 (6%)	0.69	0.58
**Treatment Response Among All Children**				
Weight gain at week 1 [g/kg/day], mean (SD)				
Overall	9 (8)	11 (8)	< .001	< .001
Boys	9 (8)	11 (8)	< .001	< .001
Girls	8 (8)	12 (7)	< .001	< .001
Weight gain at week 2 [g/kg/day], mean (SD)	6 (4)	8 (10)	< .001	< .001
MUAC gain at week 1 [mm/day], mean (SD)	5 (5)	5 (5)	0.68	0.60
MUAC gain at week 2 [mm/day], mean (SD)	7 (6)	7 (6)	0.44	0.01
**Treatment Response Among Recovered Children**				
Weight gain at week 1 [g/kg/day], mean (SD)				
Overall	10 (7)	13 (7)	< .001	< .001
Boys	10 (7)	13 (7)	< .001	< .001
Girls	10 (7)	14 (7)	< .001	< .001
Weight gain at week 2 [g/kg/day], mean (SD)				
Overall	7 (4)	9 (11)	< .001	< .001
Age < 24 months	7 (4)	9 (14)	< .001	< .001
Age ≥ 24 months	9 (4)	10 (4)	0.21	0.30
MUAC gain at week 1 [mm/day], mean (SD)	6 (5)	6 (4)	0.38	0.95
MUAC gain at week 2 [mm/day], mean (SD)	9 (6)	9 (6)	0.47	0.02
Length of stay [days], mean (SD)	29 (10)	30 (11)	0.50	0.98
**Type of Discharge**				
Recovered	1194 (64%)	349 (66%)	0.40	0.47
Transferred	650 (35%)	169 (32%)	0.23	0.16
Transferred due to clinical complications	185 (10%)	52 (10%)	0.98	0.90
Transferred due to weight loss or lack of weight gain	481 (26%)	123 (23%)	0.25	0.26
Died	10 (1%)	3 (1%)	0.94	0.90
Defaulted	15 (1%)	9 (2%)	0.08	0.12

*P value from linear, binomial or ordinal regression adjusted for parent trial intervention group.

**P value from linear, binomial or ordinal regression adjusted for parent trial intervention group, age (< or ≥ 24 months), sex, and stunting (HAZ < or ≥ -2).

To describe potential heterogeneity within children who would be included in a MUAC < 115 mm program, the second analysis compared children with both MUAC < 115 mm and WHZ < -3 (n = 940) vs. children with MUAC < 115 mm and WHZ ≥ -3 (n = 929) (**Table 2**). Children with MUAC < 115 mm and WHZ < -3 were older and male; both groups were similar in terms of clinical characteristics on admission, except for a greater prevalence of diarrhea (34% vs. 29%) and bacteremia (5% vs. 2%) among children with MUAC < 115 mm and WHZ < -3. Compared to children with MUAC < 115 mm and WHZ ≥ -3, children with both MUAC < 115 mm and WHZ < -3 were less likely to recover, particularly children < 24 months of age (71% vs. 53%), and were more likely to be transferred to inpatient care due to lack of weight gain or weight loss. Immediate weight gain was greater among children with both MUAC < 115 mm and WHZ < -3 compared to children with MUAC < 115 mm and WHZ ≥ -3, though length of stay was greater.

**Table 2:** Demographic and clinical characteristics on admission and program outcomes of children eligible for nutritional treatment using a MUAC-only admission criterion, by MUAC- and WHZ-defined admission criteria.

Child characteristics	MUAC < 115 mm and WHZ ≥ -3	MUAC < 115 mm and WHZ < -3	P*	P**
N (%)	929 (50%)	940 (50%)		
**Demographic characteristics on admission**				
Age, n (%)			< .001	< .001
6–11 months	446 (48%)	316 (34%)		
12–23 months	368 (40%)	415 (44%)		
24–59 months	115 (12%)	209 (22%)		
Females, n (%)	652 (70%)	410 (44%)	< .001	< .001
**Anthropometric status on admission**				
WHZ, mean (SD)				
Overall	-2.46 (0.44)	-3.53 (0.43)	< .001	< .001
Age < 24 months	-2.43 (0.45)	-3.52 (0.42)	< .001	< .001
Age ≥ 24 months	-2.64 (0.33)	-3.59 (0.45)	< .001	< .001
MUAC (mm), mean (SD)				
Overall	112 (28)	110 (42)	< .001	< .001
Boys	112 (26)	111 (39)	< .001	< .001
Girls	111 (29)	109 (45)	< .001	< .001
HAZ, mean (SD)	-3.02 (1.17)	-3.06 (1.27)	0.57	0.06
HAZ <-2, n (%)	755 (81%)	754 (80%)	0.54	0.004
**Clinical characteristics on admission**				
Diarrhea in last 24 h	271 (29%)	318 (34%)	0.03	0.08
Nasal discharge	204 (22%)	222 (24%)	0.39	0.25
Cough	151 (16%)	157 (17%)	0.81	0.72
Vomiting	57 (6%)	55 (6%)	0.80	0.80
Tachypnea	17 (2%)	10 (1%)	0.17	0.12
Fever [Temperature ≥ 38.5⁰ C]	49 (5%)	52 (6%)	0.79	0.54
Anemia [Hb < 11 g/dL]	697 (75%)	677 (72%)	0.15	0.02
Malaria positive	531 (57%)	509 (54%)	0.19	0.03
**Laboratory Findings**				
Bacteremia	10 (2%)	19 (5%)	0.04	0.10
Enterobacteriaceae	8 (2%)	18 (5%)	0.02	0.07
Bacteriuria	8 (2%)	14 (5%)	0.13	0.12
Enterobacteriaceae	8 (2%)	13 (4%)	0.18	0.15
Bacterial gastroenteritis	36 (8%)	53 (13%)	0.01	0.01
Enterobacteriaceae	22 (5%)	31 (8%)	0.07	0.09
*Campylobacter spp*.	16 (4%)	26 (7%)	0.05	0.03
**Treatment Response Among All Children**				
Weight gain at week 1 [g/kg/day], mean (SD)				
Overall	7 (7)	10 (9)	< .001	< .001
Boys	8 (7)	10 (8)	< .001	0.002
Girls	7 (7)	11 (10)	< .001	< .001
Weight gain at week 2 [g/kg/day], mean (SD)				
Overall	5 (4)	7 (5)	< .001	< .001
Boys	6 (4)	7 (4)	0.004	0.03
Girls	5 (4)	7 (5)	< .001	< .001
MUAC gain at week 1 [mm/day], mean (SD)				
Overall	5 (5)	5 (5)	0.85	0.25
Boys	5 (5)	5 (5)	0.06	0.03
Girls	4 (5)	5 (5)	0.37	0.72
MUAC gain at week 2 [mm/day], mean (SD)				
Overall	7 (6)	7 (6)	0.28	0.005
Boys	8 (6)	7 (6)	0.006	0.001
Girls	7 (6)	7 (6)	0.88	0.37
**Treatment Response Among Recovered Children**				
Weight gain at week 1 [g/kg/day], mean (SD)				
Overall	8 (7)	12 (8)	< .001	< .001
Boys	9 (6)	11 (7)	< .001	0.001
Girls	8 (7)	13 (8)	< .001	< .001
Weight gain at week 2 [g/kg/day], mean (SD)				
Overall	6 (4)	8 (4)	< .001	< .001
Boys	7 (4)	8 (4)	< .001	< .001
Girls	6 (4)	9 (4)	< .001	< .001
Length of stay [days], mean (SD)				
Overall	26 (8)	33 (11)	< .001	< .001
Boys	26 (7)	33 (11)	< .001	< .001
Girls	27 (9)	32 (11)	< .001	< .001
MUAC gain at week 1 [mm/day], mean (SD)				
Overall	6 (5)	6 (5)	0.90	0.23
Boys	6 (5)	5 (5)	0.03	0.02
Girls	5 (5)	6 (5)	0.29	0.61
MUAC gain at week 2 [mm/day], mean (SD)				
Overall	9 (6)	9 (5)	0.87	0.09
Boys	10 (6)	9 (5)	0.01	0.003
Girls	8 (6)	9 (5)	0.26	0.77
**Type of Discharge**				
Recovered				
Overall	664 (71%)	530 (56%)	< .001	< .001
Age < 24 months	574 (71%)	385 (53%)	< .001	< .001
Age ≥ 24 months	90 (78%)	145 (69%)	0.09	0.05
Transferred	252 (27%)	398 (42%)	< .001	< .001
Transferred due to clinical complications	79 (9%)	106 (11%)	0.05	0.06
Transferred due to weight loss or lack of weight gain	201 (22%)	280 (30%)	< .001	< .001
Died	4 (0%)	6 (1%)	0.54	0.51
Defaulted	9 (1%)	6 (1%)	0.43	0.37

*P value from linear, binomial or ordinal regression adjusted for parent trial intervention group.

**P value from linear, binomial or ordinal regression adjusted for parent trial intervention group, age (< or ≥ 24 months), sex, and stunting (HAZ < or ≥ -2).

## Discussion

Our results show that children identified for therapeutic feeding with MUAC < 115 mm were clinically similar on admission to children currently eligible for treatment but excluded using the MUAC-based admission criterion: specifically, we found no difference between groups in terms of clinical and laboratory characteristics and program outcomes evaluated in this study population. Among children with MUAC < 115 mm, we found that those with both MUAC < 115 mm and WHZ < -3 presented with a more severe clinical condition, demonstrated by increased prevalence of diarrhea and bacteremia, and did not respond as well to treatment, evidenced by a lower risk of nutritional recovery and an increased risk of transfer to inpatient care, compared to children admitted with MUAC < 115 mm and WHZ ≥ -3.

Debate and interest surrounding the use of MUAC-only in nutritional programming continues, but a shift from therapeutic feeding programs admitting children using MUAC < 115 mm and/or WHZ < -3 to a new model admitting children using MUAC only remains complicated by limited information regarding the clinical profile and response to treatment of children selected by MUAC vs. WHZ. It has been shown that a single MUAC-based cut-off (e.g. independent of age and sex) is more likely to identify SAM children who are younger, female and stunted [19–21]. It has been suggested that such a demographic and anthropometric shift in the patient profile selected with a single MUAC-based admission criterion may appropriately identify children that are more vulnerable or in need of nutritional rehabilitation. We compared children who would be included in a MUAC-based program (e.g. MUAC < 115 mm as the single anthropometric criterion) with children who are currently eligible for treatment but would be excluded from a MUAC-based program (MUAC ≥ 115 mm and WHZ < -3) in terms of a broad range of clinical, and laboratory characteristics, as well as program outcomes, to better understand the operational implications of applying a MUAC-only anthropometric criterion for admission to therapeutic feeding in rural Niger. Our findings suggest the single criterion of MUAC < 115 mm for admission to therapeutic feeding identified few important differences between groups in this context. However, among those children who would be accepted into a MUAC-only program, we found children with both MUAC < 115 mm and WHZ < -3 were more likely to be clinically unwell at admission, indicated by increased diarrhea and bacteremia, and achieved poor program outcomes. These findings suggest that where MUAC < 115 mm is used as the single anthropometric criterion for admission to therapeutic feeding, the combination of anthropometric indicators may help to further target children at increased risk or need of additional follow-up. An upward adjustment of a single MUAC threshold above 115 mm could also be applied to increase sensitivity to select more children at high risk, but such adjustments would have important programmatic implications, including increased workload and costs, and should be context-specific.

We noted significantly greater initial weight gain (e.g. at week 1 and week 2) among children with MUAC ≥ 115 mm and WHZ <- 3 children compared to children with MUAC < 115 mm and considered several potential mechanisms or explanations of this finding. First, greater initial weight gain may reflect a greater need for nutritional therapy among children with MUAC ≥ 115 mm and WHZ <- 3. The rate of weight gains observed in this group is not inconsistent with mean weight gain observed during recovery of severely malnourished children in inpatient settings (7.8 to 10 g/kg/day, [26]) and early community-based therapeutic programs (3 to 6.8 g/kg/day, [27]). In this study population, MUAC ≥ 115 mm and WHZ <- 3 may characterize a sub-group of children with a pathophysiological condition rapidly corrected and responsive to rapid weight gain, or conversely, MUAC may characterize a sub-group of children suffering from more severe metabolic impairments that limit rapid tissue restoration. Second, it is possible the greater initial weight gain among children with MUAC ≥ 115 mm and WHZ <- 3 reflects rapid water repletion associated with correction of moderate dehydration not measured in this study. The greater effect of dehydration on WHZ assessment, compared to MUAC, and the inadequacy of WHZ to assess nutritional status of children with diarrhea or dehydration was been noted elsewhere [28, 29].

With the hypothesis that differences among children selected by MUAC < 115 mm vs. MUAC ≥ 115 mm and WHZ <- 3 may further differ by age, sex and stunting, we investigated potential effect modification by these factors. A recent study from McDonald et al. importantly highlighted an increased risk of mortality with multiple anthropometric deficits (WHZ < -2, HAZ < -2 and weight-for-age Z <-2), compared to single or no anthropometric deficits [30]. The contribution of anthropometric deficit defined by MUAC < 115 mm, however, was not considered in this study. In the inpatient study from Kenya, Berkley et al. found no difference in the risk of death between children identified as SAM by MUAC only vs. those identified as SAM by WHZ only, but those children identified by both criteria had an increased risk of mortality [19]. We, however, found no evidence for worse treatment outcomes or response among children with HAZ < -2 and WHZ < -3. Our results do suggest that other clinical differences in children selected by low MUAC and/or low WHZ may be more pronounced in particular subgroups defined by age, sex or stunting. Identification of such subgroups can provide a more precise understanding of how anthropometric criteria may be selecting different profiles of vulnerable children.

Published data on potential variability among children selected by different anthropometric criteria remains very limited to date. The most detailed study available was done in 1999–2002 among 8,190 children hospitalized in rural Kenya [19]. This study similarly suggested that MUAC was more likely to identify severely malnourished children that were younger, female, and stunted. It also showed that mortality risk was similar in children with MUAC < 115 mm and children with MUAC ≥ 115 mm and WHZ <- 3, yet higher in children presenting both criteria at admission. Our results similarly suggest a higher vulnerability in children with both criteria compared with children with MUAC < 115 mm and WHZ ≥ -3. In comparing the clinical features of the children identified by MUAC < 115 mm only and by WHZ < -3 only, this study in Kenya also reported higher prevalence of seizures during current illness and impaired consciousness in low WHZ-only children but fewer other clinical signs (including cough, diarrhea, sub-costal indrawing and anemia). The comparison of these findings with the findings from our outpatient population in Niger is, however limited, given the specific definition of anthropometric groups for comparison and differences between the inpatient vs. outpatient study populations.

Routine data collected from two Médecins Sans Frontières (MSF) therapeutic feeding programs have recently been analyzed to explore this subject. In Burkina Faso, Goossens et al. analyzed data from 24,792 children admitted to a therapeutic feeding program using MUAC ≤ 118 mm as the sole anthropometric admission criterion. This study showed greater weight gain associated with lower MUAC at admission [12]. Program records from 2,205 children admitted to a therapeutic feeding program in South Sudan were also analyzed to compare children who would have been admitted to a MUAC-only program to those who would have been excluded from a MUAC only program but are included under current WHO guidelines. Like in our study, MUAC-eligible children were younger and more likely to be female. However, they were more severely malnourished and at a greater risk of death, but more responsive to treatment than those in the low WHZ-only group.(24) Although the tall and thin body shape of some pastoralist populations, as found in the latter South Sudan program, is known to influence the performance of different anthropometric indicators, the comparison of demographic characteristics from Niger are consistent with those reported by Grellety et al. from South Sudan.

This study is the first to date to compare the clinical and laboratory characteristics associated with the type of anthropometric diagnosis among a large population of children with uncomplicated SAM children admitted to an outpatient nutritional program. Characterization of individual vulnerability was informed by the reliable assessment of clinical and laboratory characteristics at admission, as well as through a careful follow-up over the course of the treatment. This study offers new information to extend current evidence on the potential implications of moving towards MUAC as the sole anthropometric admission criterion for therapeutic feeding.

This study does, however, have some limitations. In particular, laboratory analyses did not include parasitological or virological exam or confirmation of malaria infection. The prevalence of bacteriological infection and mortality were low, limiting the interpretation of these results. Additionally, this analysis draws from data collected during a randomized trial among children presenting to a health facility with SAM and receiving treatment. The study population does not reflect an untreated or general population, or a nutritional program that draws its participants from community-based screening and referral. These data therefore do not allow a clear conclusion regarding the use of using MUAC-only based criterion among untreated children in the community.

Therapeutic feeding programs must aim to target and treat malnourished children that are the most vulnerable and at the highest risk of death, but programs are often challenged to do so in highly resource-constrained settings. It was believed that a single MUAC-based anthropometric admission criterion, in addition to its operational advantages, would identify the most vulnerable children in need of treatment. Our study suggests that a single anthropometric admission criterion of MUAC < 115 mm identified younger and more female children but did not well differentiate children in terms of other measures of vulnerability. Children with MUAC < 115 mm and children with MUAC ≥ 115 mm and WHZ < -3 in rural Niger displayed similar characteristics in terms of their clinical and laboratory profile on admission. An upward adjustment of the MUAC threshold could yield a more sensitive criterion for admission to select children at high risk but would have important programmatic implications. For MUAC to be considered as a single anthropometric admission criterion for therapeutic feeding, experience with various MUAC-based thresholds and supporting evidence on the clinical characteristics and needs of children selected with each should be further evaluated in a broad range of contexts.

## Supporting Information

S1 Trial Protocol (PDF)
