# Supplementary material for: Comparison of Clinical Characteristics and Treatment Outcomes of Children Selected for Treatment of Severe Acute Malnutrition Using Mid Upper Arm Circumference and/or Weight-for-Height Z-Score
Source: PLoS One. 2015 Sep 16;10(9):e0137606. doi: 10.1371/journal.pone.0137606 (PMC4574398; doi:10.1371/journal.pone.0137606)
Supplement: S1 Trial Protocol — (PDF) [file pone.0137606.s001.pdf]

L'effet de l'utilisation systématique d'un antibiotique dans le traitement en ambulatoire des enfants sévèrement malnutris sans complication

District sanitaire de Madarounfa
Maradi, Niger 2012-2013

Juillet 2012

Centre Collaborateur de l'OMS
pour la Recherche en Epidémiologie
et la Réponse aux Maladies Emergentes

TÉLÉPHONE : 00 33 (0)1 40 21 28 48
FAX : 00 33 (0)1 40 21 28 03
E-MAIL : EPIMAIL@EPICENTRE.MSF.ORG
WEB : HTTP://WWW.EPICENTRE.MSF.ORG

ASSOCIATION LOI 1901.

PARTICIPANTS A L'ETUDE

**Titre**	L'effet de l'utilisation systématique d'un antibiotique dans le traitement en ambulatoire des enfants sévèrement malnutris sans complication
**Promoteur**	Epicentre
**Principal investigateur**	Sheila Isanaka<br>Epicentre<br>8 rue Saint Sabin<br>Paris 75011, France<br>Email : sheila.isanaka@epicentre.msf.org
**Co-investigateurs**	Rebecca Grais, Epicentre<br>Email : rebecca.grais@epicentre.msf.org<br><br>Céline Langendorf, Epicentre<br>Email : celine.langendorf@epicentre.msf.org<br><br>Fatou Berthe, Epicentre<br>Email : fatou.berthe@epicentre.msf.org<br><br>Mahaman Bachard Iro, FORSANI<br>Email: forsani-ong@yahoo.fr<br><br>Boureima Hassane, District sanitaire de Madarounfa<br>Email : hassane_b@yahoo.fr
**Equipe de développement du protocole**	Lynda Woi Messé, Epicentre<br>Saif Abdallah, Epicentre<br>Emmanuel Baron, Epicentre<br>Mandjouh Touré, Epicentre<br>Antoine Andremont, Hôpital Bichat Claude-Bernard<br>Eric Adehossi, MSP Niger<br>Guero Maimouna, Direction de la Nutrition, MSP Niger<br>Manzo Laouali, Direction régionale de Maradi, MSP
**Partenaires opérationnels**	District Sanitaire de Madarounfa, Région de Maradi, Niger<br><br>FORSANI
**Partenaire financier**	Médecins Sans Frontières-France

ABREVIATIONS

ARC	Assistant de Recherche Clinique
ASC	Agent de Santé Communautaire
ATPE	Aliments Thérapeutiques Prêts à l'Emploi
CCP	Comité de Protection des Personnes
CRENAM	Centre de Récupération et d'Education Nutritionnelle Ambulatoire pour Modérés
CRENA	Centres de Récupération et d'Education Nutritionnelle Ambulatoires
CRENI	Centre de Récupération et d'Education Nutritionnelle Intensif
C3G	Céphalosporines de Troisième Génération
E-BLSE	Entérobactéries avec une Béta-Lactamase à Spectre Étendu
FORSANI	Forum Santé Niger
GEE	Équations d'estimations généralisées
IM	Intra-Musculaire
IV	Intra-Veineux
MAG	Malnutrition Aiguë Globale
MAM	Malnutrition Aiguë Modérée
MAS	Malnutrition Aiguë Sévère
mL	Millilitres
MSF-F	Médecins sans Frontières-France
OMS	Organisation Mondiale de la Santé
STGG	Skim milk (lait écrémé), Tryptone, Glucose, Glycerin
UNICEF	Fonds des Nations Unies pour l'Enfance
VIH	Virus de l'Immunodéficience Humaine
WHZ	Z-score Poids-Taille

TABLE DES MATIERES

INTRODUCTION…………………………………………………………………………………………….5

1 CONTEXTE………………………………………………………………………………………….5

1.1 La prise en charge de la malnutrition aigue……………………………………………………5

1.2 Les infections dans la MAS………………………………………………………………………….6

1.3 Justification de l'étude…………………………………………………………………………………8

2 BUTS ET OBJECTIFS DE L'ETUDE……………………………………………………………..9

2.1 But de l'étude…………………………………………………………………………………………. 9

2.2 Objectif principal…………………………………………………………………………………….10

2.3 Objectifs secondaires………………………………………………………………………………..10

3 METHODES…………………………………………………………………………………………………..10

3.1 Site de l'étude……………………………………………………………………………………………10

3.2 Population d'étude…………………………………………………………………………………….11

3.3 Intervention de l'étude………………………………………………………………………………12

3.4 Affectation randomisée, occultation, attribution…………………………………………12

3.5 Taille de l'échantillon et échantillonnage……………………………………………………13

3.6 Soins standards…………………………………………………………………………………………13

3.7 Analyses bactériologiques en laboratoire……………………………………………………14

3.8 Saisie, gestion et contrôle de la qualité des données…………………………………..16

3.9 Analyses statistiques………………………………………………………………………………….17

4 PROCEDURES DE L'ÉTUDE……………………………………………………………………..17

4.1 Période de l'étude……………………………………………………………………………………..17

4.2 Procédures de l'étude………………………………………………………………………………..19

5 CONSIDERATIONS ETHIQUES……………………………………………………………………24

6 PARTENARIATS………………………………………………………………………………………….25

7 CONSIDERATIONS ADMINISTRATIVES ET LOGISTIQUES………………………….25

8 REFERENCES……………………………………………………………………………………………28

ANNEXES……………………………………………………………………………………………………..31

INTRODUCTION

Dans la prise en charge des patients hospitalisés atteints de malnutrition aiguë sévère (MAS), l'utilisation systématique des antibiotiques est recommandée pour améliorer la réponse nutritionnelle à l'alimentation et réduire la mortalité [1]. Les infections bactériennes concomitantes compliquent souvent les cas avancés de MAS, et le risque d'infection nosocomiale pendant le traitement dans les services hospitaliers ou les unités de récupération nutritionnelle peut être important, ce qui suggère un bénéfice potentiel de la prescription systématique d'antibiotiques dans ce contexte.

En 2006, une approche à base communautaire pour la prise en charge de la MAS a été adoptée. Ainsi les enfants atteints de MAS sans complication sont traités à domicile, avec des Aliments Thérapeutiques Prêts à l'Emploi (ATPE), alors que les soins hospitaliers sont réservés aux cas avec complications médicales [2]. Cette approche, qui implique une mobilisation accrue de la communauté, un dépistage actif des cas et l'introduction des Standards de Croissance Infantile de l'Organisation Mondiale de la Santé (OMS) [2], a entraîné des changements importants dans le profil des enfants sévèrement malnutris pris en charge aujourd'hui.

Les enfants sévèrement malnutris traités en ambulatoire ont souvent moins de complications médicales et un meilleur statut nutritionnel (notamment meilleur rapport poids-taille) que les enfants traités en milieu hospitalier. Cependant, la recommandation de l'utilisation systématique d'antibiotiques pour la prise en charge hospitalière a été étendue à la prise en charge communautaire.

Nous n'avons actuellement pas suffisamment de preuves pour appuyer l'utilisation systématique des antibiotiques en tant que composant nécessaire du traitement ambulatoire des enfants atteints de MAS sans complication. Avec le profil potentiellement différent de ces enfants traités en ambulatoire et compte tenu de l'émergence de résistances aux antibiotiques, il est important de mettre en évidence les bénéfices, s'il y en a, de l'utilisation systématique des antibiotiques dans le traitement ambulatoire des enfants atteints de malnutrition sévère.

1. CONTEXTE

1.1 La prise en charge de la malnutrition aiguë

La malnutrition aiguë [Z-score poids-taille (WHZ) < -2] affecte environ 65 millions d'enfants (13%) de < 5 ans dans le monde et contribue de façon importante à la mortalité infantile et au fardeau général des maladies [3]. La malnutrition aiguë est le résultat d'une combinaison de facteurs, notamment le régime alimentaire inapproprié et les infections. Bien que les deux formes de malnutrition aiguë: la malnutrition aiguë modérée (MAM ; -3 ≤ score WHZ < -2) et la malnutrition aiguë sévère (MAS ; score WHZ < -3) accroissent la mortalité et la morbidité, les risques liés à la malnutrition aiguë sont directement associés à sa gravité. Par rapport aux enfants ayant un score

WHZ supérieur à -1, le risque de décès est 3 fois supérieur pour les enfants atteints de MAM, et 9,4 fois supérieur pour les enfants atteints de MAS [4].

La prise en charge de la malnutrition aiguë exige une intervention spécialisée. Le protocole de l'OMS (1999) pour la prise en charge de la MAS [1] recommande un traitement à l'hôpital, avec une alimentation à base de lait fortifié, en deux phases (stabilisation et récupération) et l'administration systématique de médicaments (antibiotiques, antihelminthique et, si nécessaire, vitamine A, vaccination contre la rougeole et antipaludiques) afin de traiter et de prévenir les complications. Pendant la phase de stabilisation (2 à 7 jours), les complications potentiellement mortelles sont traitées et les anomalies métaboliques sont corrigées. Quand l'enfant retrouve l'appétit, la phase de récupération (2 à 4 semaines) peut commencer avec une alimentation intensive, afin d'obtenir un gain de poids. La standardisation des protocoles de prise en charge de la MAS a permis de réduire la mortalité. Néanmoins, une approche exclusivement hospitalière est limitée par les besoins en personnel médical qualifié, par la capacité en lits, par le risque d'infections nosocomiales et par les coûts élevés des indemnités accordées aux mères accompagnantes.

En 2006, après une accumulation de données opérationnelles et épidémiologiques confirmant l'acceptabilité et l'efficacité de la prise en charge communautaire de la MAS, l'OMS, le Programme Alimentaire Mondial, le Fonds des Nations Unies pour l'Enfance (UNICEF) et le Comité Permanent de l'Organisation des Nations Unies sur la Nutrition, ont approuvé la prise en charge communautaire de la malnutrition aiguë [5]. Contrairement à l'approche hospitalière, l'approche à base communautaire permet aux enfants diagnostiqués avec une MAS sans complication d'être traités à domicile, avec l'administration d'ATPE. Le suivi clinique et le traitement sont dispensés dans des centres ambulatoires de façon hebdomadaire ou bihebdomadaire. Basés sur les principes du traitement hospitalier, les médicaments de routine (notamment antibiotique, anti-helminthique et, si nécessaire, vitamine A, vaccination contre la rougeole et antipaludiques), sont administrés à tous les enfants atteints de MAS et traités en ambulatoire.

Dans l'approche à base communautaire, selon le protocole de traitement de l'OMS, le traitement hospitalier est réservé aux enfants atteints de MAS compliquée par une symptomatologie ou une maladie potentiellement mortelle (par exemple anorexie, œdème, fièvre, hypothermie, vomissements, détresse respiratoire, anémie sévère, infection cutanée, déshydratation ou troubles de la conscience). La proportion d'enfants souffrant de MAS qui se présentent tôt, avant le développement des complications, et qui peuvent être soignés en ambulatoire est de 70% à 90% [6,7]. Ce résultat dépend de l'efficacité de la mobilisation communautaire, du dépistage actif des cas et du contexte sanitaire dans lequel fonctionne le programme nutritionnel.

1.2 Les infections dans la MAS

Importance et profil de l'infection dans la MAS. L'interaction entre la malnutrition et l'infection est bidirectionnelle. L'infection non seulement contribue, mais est aussi une conséquence de la malnutrition [8]. Les données existantes suggèrent que les enfants atteints de malnutrition ont souvent des infections dues à des pathogènes similaires à ceux des enfants non-malnutris [9].

Néanmoins, chez les enfants malnutris, les infections semblent plus fréquentes, plus graves et plus longues [9].

Les signes d'infection sont souvent difficiles à détecter chez les enfants malnutris. Contrairement aux enfants non-malnutris qui répondent à l'infection par la fièvre et l'inflammation, les enfants malnutris atteints d'infections sévères peuvent être seulement apathiques ou somnolents [1]. Bien qu'asymptomatiques, beaucoup de cas de MAS sont compliqués par une infection bactérienne, notamment infection respiratoire, infection urinaire et infection gastrointestinale [10]. Les études épidémiologiques ont rapporté une prévalence d'infection bactérienne entre 8 et 30% chez les enfants hospitalisés atteints de MAS, avec une grande diversité de bactéries gram-positif et gram-négatif [11,12,13,14,15,16]. Cependant, la prévalence des pathogènes et le risque d'infection des enfants malnutris sévères non compliqués pris en charge en ambulatoire restent peu décrits.

Directives et pratiques actuelles pour le traitement de l'infection chez les cas de MAS. Le protocole de l'OMS (1999) recommande le traitement précoce des infections bactériennes avec des antibiotiques efficaces, afin d'améliorer la réponse nutritionnelle à l'alimentation, prévenir le choc septique et réduire la mortalité. Ce protocole déclare que : "Les infections bactériennes étant fréquentes et difficiles à détecter, tous les enfants atteints de malnutrition sévère doivent recevoir systématiquement des antimicrobiens à large spectre dès leur admission. [1]."

Tout en reconnaissant que les profils locaux d'antibiorésistances des bactéries pathogènes, ainsi que la disponibilité et le coût des antimicrobiens détermineront la stratégie, l'OMS suggère le schéma thérapeutique suivant :

- pour tous les enfants souffrant de malnutrition sévère sans signe apparent d'infection et sans complication : cotrimoxazole par voie orale 2 fois par jour pendant 5 jours.

- à tous les enfants souffrant de malnutrition sévère avec complications (choc septique, hypoglycémie, hypothermie, infections cutanées, infections des voies respiratoires ou urinaires, ou si les enfants semblent léthargiques ou malingres : ampicilline à 50mg/kg intra-musculaire (IM) ou intra-veineux (IV) toutes les 6 heures pendant 2 jours, suivie par l'amoxicilline à 15mg/kg par voie orale toutes les 8 heures pendant 5 jours (si l'amoxicilline n'est pas disponible, administrer de l'ampicilline, 25mg/kg par voie orale toutes les 6 heures) et de la gentamicine à 7,5 mg/kg IM ou IV une fois par jour pendant 7 jours.

Bien que le cotrimoxazole soit suggéré en utilisation systématique dans le traitement hospitalier de la MAS, il ne semble pas qu'il y ait un accord clair sur le traitement antibiotique le plus approprié dans ce cas. L'amoxicilline est également fréquemment utilisée dans les protocoles standards, à cause de son large spectre d'activité et son faible coût.

La déclaration de consensus de 2006, qui autorisait la prise en charge communautaire de la malnutrition aiguë, recommande une courte administration d'antibiotiques pour traiter l'infection dans le cadre de la prise en charge ambulatoire de la MAS. La prise en charge ambulatoire de la MAS utilise le même schéma d'antibiotique que celui suggéré pour le traitement hospitalier.

Résistance antimicrobienne. L'émergence de la résistance aux antibiotiques est un problème complexe guidé par de nombreux facteurs, notamment l'utilisation inappropriée des antibiotiques. La prévention et le contrôle de la résistance par un usage rationnel des antibiotiques est un problème majeur de santé publique. Les bactéries résistantes provoquent des infections qui sont plus difficiles à traiter et qui exigent des antibiotiques parfois moins disponibles et plus coûteux [17].

La fréquence de la résistance aux antibiotiques varie d'un pays à l'autre, mais augmente dans le monde entier [18,19]. Des taux élevés de résistance *in vitro* à l'ampicilline et au cotrimoxazole ont été déclarés dans plusieurs pays d'Afrique [11,16,20,21,22,23]. Une étude menée en milieu hospitalier au Kenya a décrit un taux de résistance élevé aux antibiotiques couramment administrés, notamment l'érythromycine, l'ampicilline, le cotrimoxazole, le chloramphénicol et l'oxacilline [16]. Dans une étude descriptive menée chez des enfants hospitalisés atteints de MAS à Maradi, au Niger, des niveaux élevés de résistance ont été décrits parmi un certain nombre de pathogènes isolés (résistance à la pénicilline G de 94% des Staphylocoques isolés des hémocultures, résistance à l'amoxicilline et au cotrimoxazole de 80-100% des Salmonelles et *E.coli* isolés des hémocultures, et plus de 70% des Shigelles isolés des selles [24].

Ces données suggèrent que l'amoxicilline et le cotrimoxizole peuvent s'avérer moins efficaces contre les souches de bactéries isolées chez les enfants hospitalisés, à cause de la résistance.
L'importance de la résistance antimicrobienne chez les enfants malnutris pris en charge en ambulatoire n'a pas encore été bien caractérisée.

1.3 Justification de l'étude

Les directives actuelles pour la prise en charge hospitalière et ambulatoire de MAS recommandent le traitement précoce des infections bactériennes par des antibiotiques, afin d'améliorer la réponse nutritionnelle à l'alimentation, de prévenir le choc septique et de réduire la mortalité. Cependant, les développements récents dans la définition et la prise en charge de la malnutrition aiguë suggèrent le besoin de réévaluer l'utilisation systématique des antibiotiques dans le traitement ambulatoire de la MAS.

L'introduction des Normes de Croissance Infantile de l'OMS, tout en représentant une avancée importante dans la compréhension et la mesure du statut nutritionnel des enfants, a entraîné une augmentation des admissions dans les programmes nutritionnels. De plus, les enfants inclus ont des rapports poids-taille relativement plus élevés et moins de complications médicales que ceux préalablement identifiés avec la référence du Centre National pour les Statistiques en Santé [25]. De même, l'adoption de l'approche à base communautaire pour la prise en charge de la malnutrition, avec une mobilisation communautaire accrue et un dépistage actif des cas, a contribué à l'identification précoce des enfants malnutris, avant la survenue des complications. Suite à ces développements, les enfants atteints de malnutrition sévère traités actuellement en ambulatoire peuvent être très différents, en termes de nombre absolu et de caractéristiques cliniques, que les enfants sévèrement malnutris pour lesquels les précédents protocoles de prise

en charge hospitalière ont été écrits. Malgré ces différences, la recommandation de l'utilisation systématique des antibiotiques a été étendue des patients hospitalisés aux patients en ambulatoire.

Il est maintenant nécessaire de mettre en évidence le bénéfice, s'il y en a, de l'utilisation systématique des antibiotiques chez les enfants malnutris sévères traités en ambulatoire. Historiquement, l'amoxicilline et le cotrimoxizole ont été couramment utilisés dans ce but du fait de leur faible coût et de leur large spectre d'activités face à la diversité des organismes responsables d'infections sévères chez les enfants hospitalisés pour MAS. Cependant, nous avons peu d'informations sur le profil des infections des enfants atteints de MAS traités en ambulatoire, et il ne faudrait pas présumer qu'un antibiotique adapté au traitement hospitalier des enfants sévèrement malnutris le soit également pour le traitement ambulatoire. Les résultats d'une étude rétrospective chez les enfants atteints de malnutrition sévère traités en ambulatoire ne suggèrent aucune différence du taux de guérison entre un traitement par l'amoxicilline et aucun antibiotique [25].

Des données non publiées d'un récent essai clinique randomisé au Malawi suggèrent que la prescription systématique d'antibiotiques chez les enfants sévèrement malnutris traités en ambulatoire améliorerait la récupération nutritionnelle et réduirait le risque de décès[26]. Cette étude a été menée dans un contexte à forte prévalence de kwashiorkor et d'infection par le virus de l'immunodéficience humaine (VIH). L'effet de l'utilisation d'antibiotique chez les enfants sans œdème et non exposés au VIH reste donc à explorer.

Des études supplémentaires sont nécessaires pour déterminer si l'utilisation systématique des antibiotiques peut être interrompue de façon sûre chez les enfants atteints de malnutrition sévère traités en ambulatoire. Une telle démonstration pourrait permettre une prescription plus réfléchie et plus prudente des antibiotiques, tout en maintenant la récupération nutritionnelle et la prévention de la résistance aux antibiotiques.

2. BUT ET OBJECTIFS DE L'ETUDE

2.1 But

Cette étude a pour but d'évaluer l'impact de la prescription systématique d'antibiotiques dans la prise en charge ambulatoire des cas de MAS sans complication en termes de récupération nutritionnelle, de développement de complications et/ou décès, et d'émergence de résistances aux antibiotiques.

Les stratégies de prescription à comparer sont les suivantes :

- amoxicilline (80 mg/kg/jour, 2 doses par jour pendant 7 jours)
- placebo (2 doses par jour pendant 7 jours).

La prescription de l'antibiotique est en conformité avec le protocole national de prise en charge de la malnutrition en vigueur au Niger, dont la dernière révision a été effectuée en 2012 [27].

2.2 Objectif principal

Le principal objectif de cette étude est de comparer l'absence de prescription systématique d'antibiotique par rapport à une prescription systématique d'antibiotique à base d'amoxicilline en termes de récupération nutritionnelle.

2.3 Objectifs secondaires

Les objectifs secondaires de cette étude sont de comparer les groupes de traitement en termes d'hospitalisations, d'abandons, d'absences de réponse et de décès, de gain de poids, de taille et de périmètre brachial, de survenue de signes cliniques individuels évalués au cours de chaque visite et enfin de développement et de transmission d'antibiorésistances.

3. METHODES

Cette étude sera menée comme un essai randomisé, en double aveugle, contre placebo. La randomisation se fera au niveau individuel. Seuls les enfants sévèrement malnutris éligibles pour un traitement ambulatoire, et ne nécessitant pas un traitement antibiotique spécifique, seront considérés pour l'inclusion dans l'étude.

3.1 Site de l'étude

L'étude sera menée dans le district sanitaire de Madarounfa, situé dans la région de Maradi, au Niger. Le Niger est un pays enclavé du Sahel, avec une population estimée à 16 273 738 habitants en janvier 2012 par l'Institut National de la Statistique. La production alimentaire des ménages est liée à l'agriculture pluviale non irriguée, où les cultures de base telles que le millet et le sorgho sont récoltées une fois par an de septembre à octobre. Chaque année, la diminution de la quantité et de la qualité de l'alimentation pendant les mois précédant la récolte (août à octobre) est associée à l'augmentation de la malnutrition sévère chez les enfants moins de 5 ans.

La région de Maradi est beaucoup affectée par cette problématique. En effet, selon l'Enquête de Nutrition menée dans toutes les régions du Niger de mai à juin 2011 par l'Institut National de la Statistique et la Direction de la Nutrition du MSP du Niger sur un échantillon de 9221 enfants de 6 à 59 mois, la prévalence de la malnutrition aiguë globale (MAG) dans la région de Maradi était estimée à 12.2 % (10.3-14.5). Selon la même enquête, les données de MAG au niveau du pays étaient estimées à 12.3 % (11.4-13.3). Le district de Madarounfa appartenant à cette région et qui compte environ 405 000 habitants, se situe à la frontière du Nigeria. Il a l'un des taux de malnutrition le plus élevé du pays car en juin 2010, le département a été évalué à 16,7% de MAG.

Depuis 2008, l'organisation non gouvernementale Forum Santé Niger (FORSANI) soutient les soins médicaux et nutritionnels proposés aux enfants de < 5 ans dans la région de Maradi, en collaboration avec le Ministère de la Santé Publique du Niger, Médecins sans Frontières-France (MSF-F) et l'UNICEF. Le programme nutritionnel s'appuie sur une approche ambulatoire du traitement de la malnutrition, où les enfants sans complication bénéficient d'un traitement à domicile, avec distribution d'aliments thérapeutiques prêts à l'emploi. Les cas présentant des complications médicales sont référés vers les sites hospitaliers. Dans le district de Madarounfa, FORSANI soutient la prise en charge de la MAS dans 5 centres de réhabilitation et d'éducation nutritionnelle ambulatoires (CRENA) et 1 centre de réhabilitation et d'éducation nutritionnelle intensif (CRENI). L'étude sera menée dans 4 des CRENA où le soutien de FORSANI est attendu durant toute la période de l'étude (Dan Issa, Tofa, Gabi et Madarounfa).

Le district de Madarounfa a été choisi parce que:

1) c'est une zone à haute prévalence de malnutrition
2) c'est une zone où le programme nutritionnel de FORSANI est relativement bien implanté.

3.2 Population de l'étude

Les participants à l'étude seront des enfants de 6 à 59 mois nouvellement admis pour le traitement de la MAS non compliqué (périmètre brachial < 115 mm ou WHZ < -3) dans les 4 CRENA choisis comme sites d'inclusion. Une « nouvelle admission » est définie comme une admission 3 mois ou plus après la dernière visite du programme nutritionnel.

Les critères d'inclusion dans l'étude sont :

1) Age entre 6 et 59 mois
2) Périmètre brachial < 115 mm ou rapport poids-taille Z-score < -3
3) Absence d'oedèmes bilatéraux
4) Eligible pour une nouvelle admission dans le programme nutritionnel ambulatoire dans un des 4 CRENA de Madarounfa sélectionnés comme site d'inclusion
5) Résidence principale de l'enfant dans le département de Madarounfa au Niger, dans un rayon de 15 Km autour du CRENA.
6) Absence de pathologie intercurrente exigeant des soins hospitaliers [1]
7) Absence de pathologie intercurrente exigeant la prescription d'un traitement antibiotique spécifique par le médecin de l'étude
8) Consentement écrit d'un parent ou d'un responsable de l'enfant pour participer à l'étude.

[1] Conformément aux protocoles actuels des traitements, les enfants seront transférés à un centre de soins hospitalier en cas de manque d'appétit, troubles de la conscience, fièvre, vomissements, déshydratation sévère, anémie sévère, détresse respiratoire, présence d'œdème ou d'infections cutanées.

Les critères d'exclusion de l'étude sont :

1) Age < 6 mois ou > 59 mois
2) Périmètre brachial ≥ 115 mm et rapport poids-taille Z-score ≥ -3
3) Présence d'oedèmes bilatéraux
4) Résidence principale de l'enfant en dehors de la zone géographique délimitée par l'étude au Niger
5) Présence de pathologie intercurrente exigeant des soins hospitaliers [1]
6) Présence d'une pathologie intercurrente exigeant la prescription, par le médecin de l'étude, d'un traitement antibiotique spécifique, à l'admission
7) Présence d'anomalie congénitale ou de maladie chronique pouvant affecter la croissance, ou le risque d'infection
8) Prise d'un antibiotique (quel qu'il soit) dans les 7 jours précédents
9) Admission dans un programme nutritionnel pour le traitement de la MAS au cours des 3 mois précédents
10) Contre-indication et/ou hypersensibilité connue à l'amoxicilline.

3.3 Intervention de l'étude

Les stratégies de prescription de l'antibiotique systématique seront réparties par randomisation comme suit :

1) amoxicilline (80 mg/kg/jour, divisée pour 2 doses par jour pendant 7 jours)
2) placebo (2 fois par jour pendant 7 jours)

La stratégie de prescription sera attribuée au hasard au CRENA au moment de l'inclusion dans l'étude. La totalité du traitement attribué sera fournie à l'admission. La première dose sera administrée à l'enfant sous la surveillance de l'infirmier de l'étude.

3.4 Affectation randomisée, occultation, attribution

L'étude sera menée en double aveugle : les enfants, leur mère et le personnel de l'étude ignoreront la stratégie de prescription attribuée à l'enfant. Les traitements de l'étude (amoxicilline et placebo) seront aussi proches que possible en goût, couleur et aspect.

La liste d'attribution aléatoire sera créée à l'aide d'un générateur de nombre aléatoire informatisé, par un pharmacien du fabricant des médicaments utilisés dans l'étude qui ne sera pas impliqué dans la mise en œuvre de l'étude. La liste des codes utilisés pour la répartition aléatoire au sein des deux groupes, avec l'une des six lettres codées correspondant à l'un des deux groupes de l'étude, sera conservée à l'intérieur d'enveloppes opaques scellées et numérotées consécutivement et ouvertes par un médecin de l'étude par ordre de numérotation au moment de l'inclusion. L'assistant pharmacien présent sur chacun des sites distribuera les traitements de l'étude aux enfants selon le code d'attribution aléatoire révélé par le médecin de l'étude.

Le coordinateur de la base de recherche en poste à Maradi saura quels sont les codes attribués respectivement à l'amoxicilline et au placebo. Dans l'éventualité où des complications cliniques auraient lieu dans un délai de deux semaines à compter de l'inclusion dans l'étude et que la connaissance de l'administration antérieure d'antibiotiques soit nécessaire pour prendre en charge ces coomplications de manière adéquate, l'Assistant de Recherche Clinique sera en mesure de demander l'attribution aléatoire pour chacun des enfants au coordinateur sur le terrain.

3.5 Taille de l'échantillon et échantillonnage

Une taille d'échantillon de 1005 dans chaque groupe a été calculée, afin de détecter une différence de la proportion de guérison d'au moins 5%, en supposant un risque unilatéral α de 0,025, une puissance de 80%, et une proportion de guérison de base de 80%. En supposant un taux de perdus de vue à 20%, le nombre d'enfants à inclure dans chaque groupe est de 1206, soit un total de 2412 enfants.

En moyenne, la taille d'échantillon requise sera atteinte si 8 enfants sont inclus quotidiennement, 5 jours par semaine pendant 60 semaines (14 mois). Les 8 inclusions quotidiennes seront distribuées entre les 4 CRENA (Dan Issa, Gabi, Tofa et Madarounfa) selon la taille du CRENA.

Pendant la période de soudure, il est attendu que le nombre d'enfants admis dans les CRENA et remplissant les critères d'inclusion dépassera le nombre de patients requis par jour. Une procédure d'échantillonnage sera développée pendant cette période, afin de ne sélectionner que le nombre d'enfants requis.

3.6 Soins standards

Les enfants seront traités selon le protocole national en vigueur au Niger pour le traitement systématique et celui de MSF-F pour toute complication survenant durant le suivi lors de la prise en charge de MAS [7]. Les enfants seront éligibles pour l'admission dans le programme nutritionnel ambulatoire s'ils remplissent au moins l'un des critères suivants :

- périmètre brachial < 115 mm, si l'enfant mesure plus de 65cm
- WHZ < -3 selon les normes de croissance de l'OMS

Les enfants seront hospitalisés en cas de manque d'appétit, œdèmes bilatéraux, troubles de la conscience, fièvre, vomissements, déshydratation, détresse respiratoire, infections cutanées ou tout autre type de complication associée. Les œdèmes bilatéraux seront détectés par l'obtention d'un creux après une pression modérée du pouce sur le dessus du pied et sur l'extrémité inférieure du tibia, pendant 3 secondes (« signe du godet »).

A l'admission, tous les enfants recevront en systématique :

- une forte dose de vitamine A en capsule (100 000 UI pour les enfants < 4 kg ; 200 000 UI pour les enfants de 4-8 kg ; et 400 000 UI pour les enfants ≥ 8 kg) ;
- une dose unique d'acide folique 5mg en comprimé

- un traitement antihelminthique (200 mg d'albendazole pour les enfants < 8 kg ; 400 mg pour les enfants ≥8 kg) ;
- une dose du vaccin contre la rougeole, si c'est nécessaire, (par exemple vaccination à l'admission pour les enfants n'ayant pas de certificat de vaccination, ou à 9 mois pour les enfants admis à l'âge de 6 à 8 mois).

La présence du *Plasmodium falciparum* (infection palustre) sera recherchée chez tous les enfants à l'inclusion et en cas de fièvre pour les autres visites au moyen d'un test rapide (SD BIOLINE Malaria Ag P.f®, Standard Diagnostics, Inc., Gyeonggi-do, Republic of Korea), ainsi que la présence d'anémie, au moyen d'un photomètre à hémoglobine (Hb 301+, Hemocue®, Angelholm, Sweden). Ces 2 tests sont réalisés à partir d'un prélèvement de sang capillaire. Un traitement sera administré, si nécessaire.

Le traitement nutritionnel ambulatoire comprend d'ATPE (Plumpy'nut® de Nutriset, Malaunay, France). La quantité d'ATPE à donner par semaine correspond au poids de l'enfant, selon le protocole nationale [27].

La durée minimum du programme nutritionnel est de 3 semaines. A 3 semaines ou après, les enfants pourront sortir du programme s'ils atteignent WHZ ≥ -2 lors de deux visites consécutives[2], et un périmètre brachial > 11,5 cm. L'enfant ne devra avoir ni complication aiguë ni œdème (pendant au moins 7 jours) et avoir terminé la totalité du traitement d'antibiotique et anti paludéen à sa sortie du programme. Si le poids de l'enfant n'a pas augmenté après 2 semaines de traitement nutritionnel ou s'il y a une perte de poids > 5% entre 2 visites consécutives, l'enfant sera hospitalisé pour des examens médicaux et nutritionnels complémentaires. La durée du programme nutritionnel (ambulatoire + hospitalier) ne devrait pas dépasser 8 semaines.

3.7 Analyses bactériologiques en laboratoire

Des prélèvements de selles, d'urine, de sang et du nasopharynx seront effectués chez tous les enfants à leur inclusion dans l'étude, afin de décrire la prévalence des infections bactériennes et de l'antibiorésistance au sein des enfants souffrant de MAS non compliquée. L'antibiothérapie ne sera débutée qu'après la réalisation des prélèvements.

Tous les échantillons seront prélevés par du personnel formé et expérimenté, en respectant les mesures d'asepsie et de sécurité. Ils seront ensuite transportés le jour même du site d'inclusion vers le laboratoire Epicentre à Maradi à température ambiante ou dans un porte-vaccin réfrigéré selon le type d'échantillon.

Prélèvement sanguin. Le prélèvement sanguin pour hémoculture sera effectué grâce à une aiguille épicrânienne reliée à une seringue. Le contenu de la seringue sera ensuite transféré dans un flacon à hémoculture. Deux prélèvements sanguins seront effectués à 30 minutes d'intervalle afin de

---

[2] Selon le protocole national, les patients sortis du CRENA avec un P/ T≥ -2 Z score sont suivies au CRENAM. S'il n'y a pas de possibilité de transfert au CRENAM, le patient doit être sorti au P/T≥ -1,5 Z score.

réaliser deux hémocultures ce qui en améliorera la sensibilité et la spécificité. Le volume prélevé pour chaque hémoculture sera de 1 mL.

Prélèvement urinaire. Le prélèvement urinaire se fera grâce à une poche de recueil stérile. Un minimum de 2 mL d'urine devra être obtenu et transféré dans un tube stérile avec conservateur.

Prélèvement de selles. Les selles seront recueillies dans un pot non stérile. Un volume minimum de 2 mL sera obtenu. Un écouvillon sera ensuite plongé dans la selle puis transféré dans un tube contenant un milieu de transport semi-gélosé (Cary-Blair).

Prélèvement nasopharyngé. Un écouvillon flexible stérile sera introduit le long du nostril afin d'atteindre le nasopharynx postérieur. L'écouvillon sera ensuite plongé dans un tube contenant un milieu de transport type STGG (lait écrémé, tryptone, glucose, et glycérine).

Dès réception au laboratoire, les échantillons seront mis en culture, sur des milieux sélectifs ou non permettant de mettre en évidence puis d'identifier les bactéries pathogènes recherchées. Un antibiogramme adapté à chaque espèce bactérienne sera réalisé afin d'évaluer leur sensibilité aux antibiotiques. La gamme d'antibiotiques testés ainsi que les règles d'interprétation seront issues des recommandations du Comité de l'Antibiogramme de la Société Française de Microbiologie (2012).

Toutes ces analyses seront effectuées en aveugle par rapport à l'affectation du traitement.

Les souches bactériennes isolées au cours de l'étude seront conservées au laboratoire pour contrôle de qualité et caractérisations complémentaires éventuelles.

Les résultats confirmés de bactériémie ou de bactériurie seront rendus à l'équipe médicale de l'étude dans les 1 à 3 jours qui suivent, pour permettre un traitement approprié. L'enfant sera alors recherché à son domicile pour une évaluation clinique et il lui sera administré un traitement adapté à son état clinique.

Une étude ancillaire sur la résistance bactérienne sera également menée sur un sous-échantillon randomisé de 10% des enfants inclus et sur leur fratrie âgée de 6 à 59 mois. Il est reconnu que la flore commensale intestinale est grandement impliquée dans l'émergence et la transmission des résistances bactériennes. Parmi ces résistances, la résistance aux céphalosporines de troisième génération (C3G) reste celle la plus préoccupante en terme d'implications cliniques. Ainsi, cette étude ancillaire sera utilisée pour décrire l'émergence des résistances aux C3G dans la flore intestinale des enfants traités et la transmission de ces résistances à leur fratrie.

Les objectifs spécifiques de cette étude ancillaire seront :

1) décrire la prévalence à l'admission du portage d'Entérobactéries avec une béta-lactamase à spectre étendu (E-BLSE) (résistance aux C3G) dans les selles des enfants inclus

2) décrire l'émergence du portage de E-BLSE dans les selles des enfants inclus et de leur fratrie une semaine et quatre semaines après l'inclusion.

Durant les visites régulières à la première semaine et la quatrième semaine, les enfants sélectionnés dans l'étude ancillaire devront fournir un échantillon de selles (selon les mêmes procédures qu'à l'inclusion). De plus, une équipe Epicentre (agent de santé communautaire (ASC) et l'infirmier) se rendra au domicile des enfants sélectionnés, le jour de l'inclusion, la première semaine et la quatrième semaine, afin de prélever les selles des frères et sœurs âgés de 6 à 59 mois présents dans le ménage.

Tous les échantillons de selles collectés dans le cadre de cette étude ancillaire seront transportés au laboratoire Epicentre de Maradi le jour même. Dans ce laboratoire, la présence de E-BLSE sera déterminée par la mise en culture sur un milieu sélectif. Les espèces mises en cause ainsi que leur profil de résistance aux antibiotiques seront déterminés. De plus, une culture quantitative permettra d'évaluer la proportion de E-BLSE par rapport à l'ensemble de la flore d'Entérobactéries non-BLSE. Les souches E-BLSE isolés et suspectées de pouvoir être transmise entre les enfants inclus dans l'étude et leur fratrie seront envoyésau laboratoire de bactériologie de l'hôpital Bichat-Claude Bernard à Paris, France. Le gène de résistance aux C3G sera identifié par technique de biologie moléculaire (polymerase chain reaction, PCR). Enfin, des analyses de clonalité des E-BLSE (par rep-PCR) permettront de comparer les souches entre les enfants inclus et leurs frères et sœurs.

3.8 Saisie, gestion et contrôle de la qualité des données

Les dossiers d'observation (sous forme de questionnaires structurés) seront remplis à chaque visite. Chaque formulaire sera identifié par date, numéro d'identification de l'enfant, et signature de la personne chargée de collecter les données. Les formulaires seront placés dans des dossiers individuels jusqu'à la saisie des données.

Chaque semaine, l'ARC de l'étude vérifiera la précision des données. Les données feront l'objet d'une double saisie dans une base de données EpiData 3.1 (EpiData, Odense, Danemark). Les dossiers d'observations seront classés dans des dossiers séparés pour chaque participant de l'étude et placés dans un endroit sûr (sous clé, au sein du bureau Epicentre). Tous les documents concernant les participants, y compris les échantillons de laboratoire et les dossiers d'observation, seront identifiés avec les codes d'identification appropriés, que ce soit pour les documents papiers ou électroniques. Les noms apparaîtront uniquement sur les formulaires de consentement éclairé.

Toutes les données sur les enfants seront confidentielles et ne seront communiqués à personne en dehors de l'équipe de l'étude, sauf si l'on estime que la divulgation est nécessaire pour protéger la santé de l'enfant. La base de données électronique ne contiendra ni les noms, ni les adresses des enfants, afin de préserver leur anonymat.

3.9 Analyses statistiques

Le résultat principal est la récupération nutritionnelle. Les résultats secondaires comprennent les hospitalisations, les abandons, les absences de réponse et les décès, le gain de poids, de taille et de périmètre brachial ainsi que la survenue de signes cliniques individuels évalués au cours de chaque visite.

Toutes les données seront analysées selon le principe d'intention de traiter. Nous utiliserons un modèle de régression log-binomiale pour estimer le risque relatif et 95% des intervalles de confiance (IC) pour chacun des résultats du programme (récupération nutritionnelle, hospitalisations, abandons, absences de réponse et les décès) . Nous supposons que l'effet de l'intervention sera le plus important notamment chez les enfants avec une infection bactérienne au moment de l'admission et nous calculerons l'effet de l'intervention dans ces sous-groupes. Un test logarithmique par rangs sera utilisé afin de comparer les courbes de Nelson-Aalen pour l'incidence cumulative des hospitalisations et des décès à 12 semaines. Nous estimerons des différences moyennes en termes de gain de poids, de taille et de périmètre brachial en fonction des groupes d'intervention grâce au test de Student (prise de poids), ou à un modèle de régression linéaire ajusté pour l'anthropométrie de base (taille et périmètre brachial). Pour la survenue des signes cliniques individuels, on estimera un ratio de risque et des IC à 95% en utilisant une régression binomiale aux semaines 1 et 2 ou des équations d'estimation généralisées (GEE) avec une matrice de corrélation échangeable et des écarts-types robustes pour comparer la différence pour l'ensemble de la période du suivi.

Un analyse intérimaire après 6 mois d'inclusion sera menée afin d'assurer la sécurité des enfants inclus. Aucun ajustement ne sera fait pour des tests multiples. Toutes les valeurs de *P* seront bilatérales, avec $P < 0,05$ considéré statistiquement significatif, et aucun ajustement pour des comparaisons multiples ne sera effectué. Les analyses seront effectuées à l'aide de SAS v 9.2 (SAS Institute Inc.).

4. PROCEDURES DE L'ETUDE

4.1 Période de l'étude

L'étude durera au minimum 27 mois. La phase pilote commencera en aout 2012 et le suivi se déroulera de septembre 2012 à janvier 2014. Le calendrier de l'étude est résumé au Tableau 1.

r général de l’étude

	1	2	3	4	5	6	7	8	9	10	11	12	13	14	15	16	17	18	19	20	21	22	23	24	25	26	27
	D	J	F	M	A	M	J	J	A	S	O	N	D	J	F	M	A	M	J	J	A	S	O	N	D	J	F
cole	X	X	X	X	X																						
ité d’Ethique					X	X																					
ures					X	X	X																				
ation du					X	X	X	X																			
									X																		
										X	X	X	X	X	X	X	X	X	X	X	X	X	X				
ire*										X	X	X	X	X	X	X	X	X	X	X	X	X	X				
									X			X	X	X	X	X	X	X	X	X	X	X	X	X	X	X	
									X								X						X			X	X
t																											X

galement effectuées en cas de développement de complications cliniques nécessitant un traitement de secours.

4.2 Procédures de l'étude

Inclusions

Pour accélérer l'obtention de la taille d'échantillon voulue, les inclusions auront lieu dans 4 CRENA soutenus par FORSANI. Pendant la période de soudure (juillet – octobre) nous prévoyons que le nombre d'enfants admis aux CRENA et remplissant les critères d'inclusion dépassera le nombre d'enfants requis par jour. Une procédure d'échantillonnage sera développée pour cette période, afin de sélectionner uniquement le nombre d'enfants voulus.

Après la période de soudure (novembre-juin), il est possible que le nombre d'enfants admis aux CRENA remplissant les critères d'inclusion sera inférieur au nombre de patients requis par jour. Si c'est le cas, nous prendrons des mesures pour augmenter le nombre d'inclusions pendant les mois correspondant à la période de soudure.

Visite d'inclusion

L'inclusion s'effectuera à la première visite au CRENA. Le parcours de l'enfant du dépistage jusqu'à l'inclusion est décrit à la Figure 1. A l'arrivée au CRENA, un assistant nutritionnel vérifiera éligibilité des enfants, conformément au critère de résidence et d'âge.

- Si l'enfant vit dans le district sanitaire de Madarounfa au Niger, dans un rayon de 15 Km autour du CRENA et a entre 6 et 59 mois, les 2 assistants nutritionnels d'Epicentre prendront ses mesures anthropométriques : poids (précision 100 g), taille couchée (enfants <24 mois) ou taille debout (enfants ≥ 24 mois) avec une précision de 0,1 cm, et périmètre brachial (précision 0,1 cm).
- Si l'enfant vit en dehors de la limite géographique définie pour l'étude et / ou a moins de 6 mois, il sera orienté vers le personnel FORSANI pour le dépistage et l'ensemble de la prise en charge.
- Si l'enfant vit au Niger dans un rayon de 15 Km autour du CRENA, mais qu'il a un WHZ ≥ -3 ou un périmètre brachial ≥ 11,5 cm et / ou a plus de 59 mois, il ne sera pas admis dans le programme nutritionnel. Il sera renvoyé chez lui après explication des critères d'admission dans le programme à l'accompagnant. Au cas par cas, ces enfants pourront être dirigés vers le programme de soins pédiatriques ou le programme de vaccination, si besoin.

Si l'enfant a un rapport poids-taille < -3 et/ou un périmètre brachial < 11,5 cm, les assistants nutritionnels administreront le test d'appétit. Dès que l'enfant a terminé la dose test, il sera transféré au médecin de l'étude pour continuer la détermination des autres critères d'inclusion (antécédents médicaux, traitements reçus pendant les 7 jours précédents, traitement nutritionnel reçu pendant les 3 mois précédents, etc.). Le médecin de l'étude procédera à un examen clinique (pour déterminer la présence de complications médicales ou d'œdèmes exigeant des soins hospitaliers, les signes cliniques exigeant une prescription d'antibiotique spécifique, les anomalies congénitales ou de maladie chronique).

Figure 1. Parcours des enfants pour la sélection d’éligibilité

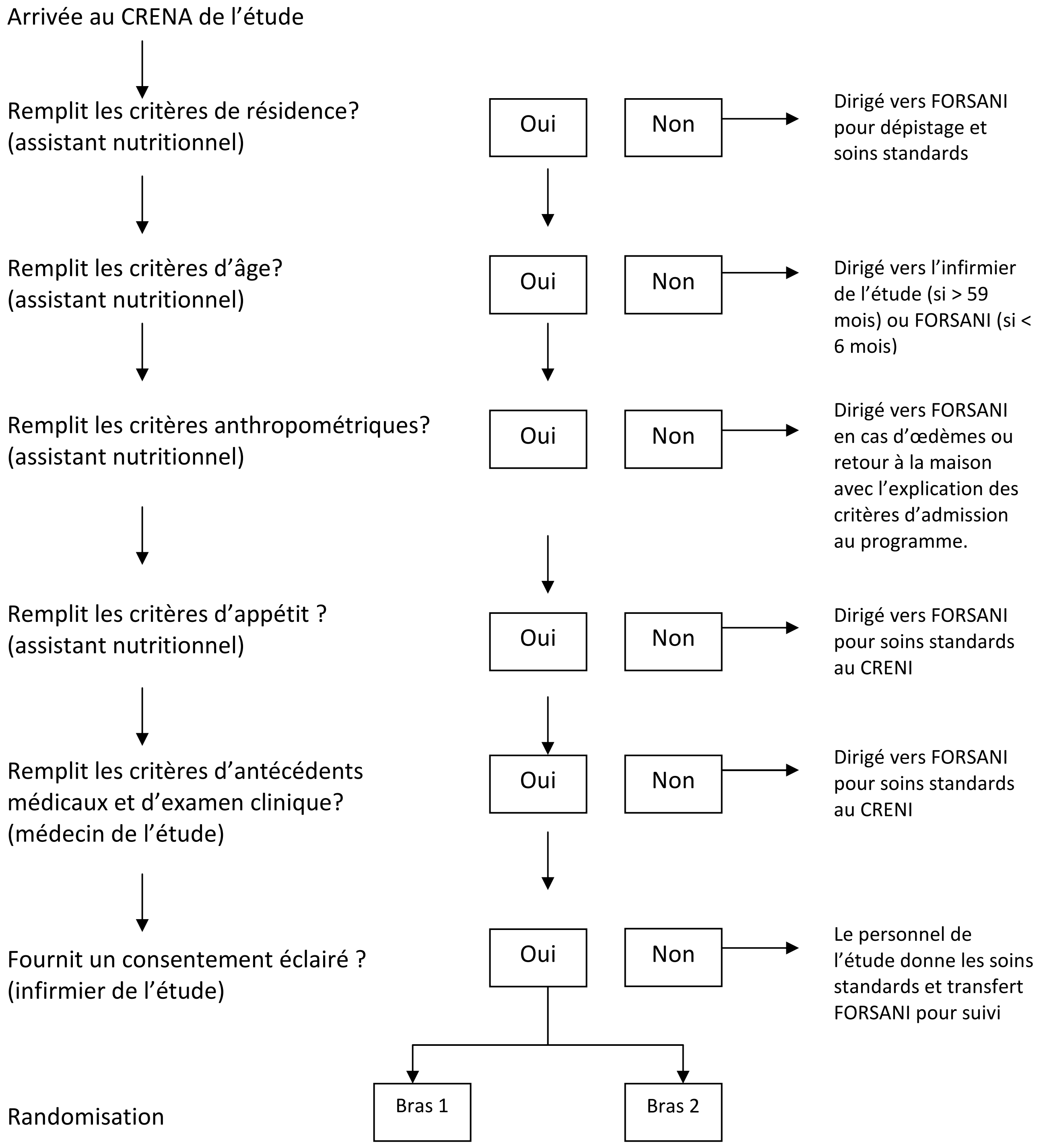

Pour les enfants éligibles devant recevoir un traitement nutritionnel ambulatoire, mais ne répondant pas à tous les critères d'inclusion de l'étude, l'infirmier de l'étude orientera l'accompagnant de l'enfant vers le circuit de prise en charge FORSANI, pour qu'il soit interrogé et consulté si nécessaire. Cette prise en charge sera assurée par les équipes de FORSANI et du Ministère de la santé travaillant au CRENA.

Les enfants nécessitant un traitement en milieu hospitalier seront référés à l'équipe de FORSANI sur le CRENA qui par la suite va assurer leur transfert au CRENI de Madarounfa qu'ils soutiennent afin d'être enregistrés et de recevoir les soins appropriés.

Pour les enfants éligibles dans l'étude et répondant à tous les critères d'inclusion, l'infirmier CRENA de l'étude indiquera à la mère les procédures de l'étude et obtiendra son consentement éclairé. Il interrogera la mère ou la personne responsable de l'enfant sur son statut socio-économique, la sécurité alimentaire du foyer, les pratiques alimentaires de l'enfant (dont allaitement), les antécédents de vaccination de l'enfant, et les maladies et traitements récents de l'enfant.

L'infirmier CRENA donnera au responsable de l'enfant une carte d'identification pour faciliter l'identification de l'enfant dans l'étude lors des suivis ultérieurs et en cas de transfert au CRENI/hospitalisation. La carte d'identificationportera le numéro d'identification, âge et sexe de l'enfant dans l'étude, sans aucune autre information d'identification.

Les prélèvements de sang, urine, selles et nasopharyngés seront ensuite collectés par l'infirmier-préleveur. Un consentement éclairé sera requis pour le test VIH chez tous les enfants. L'infirmier-préleveur réalisera le prélèvement et déterminera le statut VIH à l'aide d'un algorithme comportant la réalisation consécutive de 2 tests rapides si le premier test est positif (Determine et Uni-Gold). Les résultats discordants (et les résultats positifs pour les enfants de moins de 18 mois) seront confirmés par biologie moléculaire. Un counselling post-test adapté sera proposé et les patients positifs seront référés vers l'Hôpital Régional de Maradi pour le suivi et le traitement.

Ensuite l'infirmier CRENA accompagnera la mère et l'enfant à la pharmacie pour recevoir les médicaments du traitement systématique, la totalité de la prescription d'antibiotique attribuée par randomisation, ainsi que le traitement nutritionnel suffisant jusqu'à la visite de suivi suivante. La 1ère dose du traitement assigné (amoxicilline ou placebo) sera administrée sous la supervision de l'infirmier CRENA au moment de la dispensation. Il sera demandé à l'accompagnant de revenir au CRENA s'il estime que l'état de santé de l'enfant s'aggrave entre deux visites de suivi.

Lorsque toutes les activités d'inclusion seront terminées, l'ASC ramènera en voiture les participants de l'étude. Il notera l'emplacement exact de chaque domicile, afin de les retrouver facilement en cas de non présentation aux visites de suivi programmées. Si un enfant a été sélectionné dans l'étude ancillaire, un échantillon de selles sera collecté chez ses frères et sœurs âgés de 6 à 59 mois et présents au domicile.

A la visite d'admission et à chaque visite ultérieure, les responsables des enfants recevront une indemnité de participation à l'étude. Les mères ou responsables des enfants recevront du savon à chaque visite au CRENA, à l'exception de la dernière visite à la fin du suivi où elles recevront un pagne.

Suivi

Les visites de suivi sont programmées toutes les semaines jusqu'à la sortie du programme nutritionnel, puis une fois par mois pendant les 2 mois suivants (soit un total de 3 mois). A chaque visite, les assistants nutritionnels enregistreront les mesures anthropométriques des enfants. A chaque visite, les infirmiers CRENA de l'étude enregistreront la température et la fréquence respiratoire (à 2 reprises).

Le responsable de chaque enfant recevra un calendrier en images sur lequel il notera, à domicile, les maladies (présence de fièvre, diarrhée, vomissements, toux, écoulement nasal, troubles respiratoires, éruption cutanée, perte d'appétit), et les soins médicaux et traitements reçus depuis la visite précédente. Les infirmiers de l'étude examineront ce rapport avec le responsable de l'enfant et ils enregistreront les informations sur le dossier d'observation. Pour comprendre la consommation de l'antibiotique (traitement de l'étude) et celle du complément nutritionnel dans le ménage, il sera demandé au responsable de l'enfant de rapporter les produits non consommés lors de la visite suivante.

Le médecin de l'étude effectuera un examen physique complet, afin de déterminer le développement d'œdèmes ou de complications cliniques. Le même médecin examinera chaque enfant à toutes les visites de suivi. A n'importe quelle visite de suivi (programmée ou non), le médecin pourra remplacer le médicament à l'étude par un traitement supplémentaire si l'état clinique de l'enfant l'exige. Au moment ou les médecins de l'étude détermineront qu'un traitement supplémentaire est nécessaire, celui-ci devra être déterminé seulement en fonction de l'état clinique de l'enfant. Le choix du traitement devra prendre en considération tous les traitements disponibles et ne prendra pas en compte le traitement (amoxicilline ou placebo) qui peut avoir été attribué à l'enfant. Si le médecin détermine que tout autre médicament que l'amoxicilline est le traitement le plus approprié en fonction de l'état clinique de l'enfant, ce traitement sera donné immédiatement. Si le médecin détermine que l'amoxicilline est le traitement le plus approprié en fonction de l'état clinique de l'enfant, le médecin prendra contact avec l'ARC qui lui-même prendra contact avec le responsable de la base de recherche à Maradi pour connaitre le traitement attribué à l'enfant. En tenant compte du traitement attribué à l'enfant, l'ARC confirmera si le médecin de l'étude doit donner l'amoxicilline ou un autre traitement à l'enfant. L'attribution de codes à l'amoxicilline ou au placebo changera régulièrement, de sorte que toute supposition d'attribution faite à travers ce processus ne pourra s'étendre aux autres enfants de l'étude.

Selon les signes cliniques, des prélèvements de selles, d'urine, et de sang pourront être réalisés au moment de l'identification des complications cliniques. Les prélèvements seront transférés au laboratoire Epicentre de Maradi pour mise en culture, identification bactérienne et

antibiogramme. Les résultats seront rendus au médecin 1 à 3 jours après environ, lui permettant de réajuster les traitements et les soins, si nécessaire.

Si l'enfant développe une complication médicale exigeant des soins hospitaliers pendant le suivi de l'étude, ou s'il n'a aucun gain de poids après 2 semaines de traitement nutritionnel ou s'il y a une perte de poids > 5% entre 2 visites consécutives, il sera transféré au CRENI. Après ce transfert, le suivi de l'enfant dans l'étude sera interrompu, mais ses résultats nutritionnels et sa survie seront enregistrés à partir d'une revue du dossier d'hospitalisation de l'enfant.

Si un enfant ne se présente pas à une visite de suivi, l'ASC et l'infirmier du site de l'étude (CRENA) se rendront au domicile de l'enfant. Sur place, l'infirmier prendra le poids de l'enfant, obtiendra les antécédents de morbidité, évaluera l'état clinique de l'enfant et déterminera la raison du non respect du rendez-vous programmé.

En cas de décès, le médecin du site se rendra au domicile de la famille et l'interrogera pour connaître la cause du décès, selon les méthodes d'autopsie verbale. Si l'enfant décédé était hospitalisé, il tentera d'obtenir le dossier médical.

Mesures et définitions de la maladie
Le poids, la taille et le périmètre brachial des enfants seront mesurés selon les techniques décrites dans l'Etude de Référence Multicentrique de Croissance de l'OMS. Tous les infirmiers et tous les assistants nutritionnels de l'étude seront formés et évalués avant le début de la collecte des données. Des séances de remise à niveau seront répétées tous les mois. Nous calculerons le rapport poids-âge, le rapport taille-âge, le rapport poids-taille et les scores Z de périmètre brachial par rapport à l'âge, selon les Standards de Croissance Infantile de l'OMS 2006.

Les définitions suivantes seront utilisées pour les pathologies intercurrentes :

- diarrhée : présence d'au moins trois selles molles ou liquides par 24 heures.
- infection respiratoire supérieure : présence de toux et d'écoulement nasal purulent
- infection respiratoire inférieure : présence de toux et d'augmentation du rythme respiratoire spécifique à l'âge (>50/min pour les bébés <12 mois, et >40/min pour les enfants plus âgés).

Les apparitions de nouveaux épisodes de diarrhée et d'infections respiratoires seront prises en compte si elles se produisent au moins 72 heures après la guérison de l'épisode précédent.

Planification des procédures de l'étude. La planification des procédures de l'étude est résumée au Tableau 2.

Tableau 2. Calendrier spécifique des procédures de l'étude

Procédures	SEMAINES de VISITE pour l'étude						
	S0	S1	S2	S3	S4*	S8	S12
Evaluation initiale**	X						
Mesures anthropométriques	X	X	X	X	X	X	X
Examen clinique	X	X	X	X	X	X	X
Pathologies intercurrentes		X	X	X	X	X	X
Analyses de laboratoire ***	X	X			X		
Distribution des traitements de l'étude	X						
Distribution des aliments thérapeutiques prêts à l'emploi	X	X	X	X	X		
Distribution d'indemnité	X	X	X	X	X	X	X

* Les visites hebdomadaires de l'étude continueront jusqu'à la sortie du programme nutritionnel.

** L'évaluation initiale comprend un entretien concernant les caractéristiques sociodémographiques du foyer et les antécédents médicaux de l'enfant.

***Analyses de laboratoire également effectuées en cas de développement de complications cliniques nécessitant un traitement de secours.

5. CONSIDERATIONS ETHIQUES

L'étude appliquera les principes de la déclaration d'Helsinki et les directives règlementaires du Niger. Le recrutement des participants ne commencera pas avant que le Comité Consultatif National d'Ethique, le ministère de la Santé Publique du Niger et le Comité de Protection des Personnes (CCP), Ile-de-France, aient approuvé le protocole de l'étude. Les mères ou représentants légaux qui accepteront la participation de leur enfant devront signer un consentement éclairé, en indiquant la nature bénévole de l'étude et le droit qu'ont les participants d'interrompre le suivi à tout moment. Un comité de surveillance indépendant contrôlera l'avancement de l'étude et les évènements relatifs à l'innocuité.

L'inclusion dans l'étude sera bénévole, et se fera uniquement après le consentement éclairé écrit. Le processus de consentement consistera à présenter deux documents : une fiche d'information et un formulaire de consentement éclairé, tous deux traduits en Haoussa. L'infirmière lira à la mère la fiche d'information complète et, si elle le souhaite, la mère en recevra un exemplaire. Après avoir répondu à toutes les questions posées sur l'étude, l'infirmière de l'étude demandera à la mère de signer (ou d'apposer son empreinte digitale) le consentement éclairé. Ces mêmes procédures pour obtenir le consentement éclairé pour le test VIH seront mises en œuvre. Tous les formulaires de consentement signés seront rangés dans un endroit sûr dans le bureau Epicentre de Maradi.

L'étude sera enregistrée sur registre des essais cliniques tenu par l'Institut National de Santé des Etats-Unis (United States National Institutes of Health, www.clinicaltrials.gov).

6. PARTENARIATS

Cette étude sera menée par Epicentre, en collaboration avec FORSANI, MSF-F et le Ministère de la Santé Publique du Niger. Epicentre informera et communiquera régulièrement avec le personnel de FORSANI et du Ministère impliqué, afin que tous soient informés des procédures et de la mise en œuvre de l'étude.

Epicentre sera responsable du développement du protocole, de la supervision des procédures de l'étude, de l'analyse des données et de la rédaction du rapport pour diffusion.
L'investigateur principal sera basé à Paris. Il sera responsable du développement du protocole et suivra la mise en œuvre de l'étude. Il sera en communication régulière avec l'équipe de coordination d'Epicentre à Niamey qui supervisera la mise en œuvre de l'étude.

Un lien de l'investigateur principal et de la coordination avec les sites de l'étude se fera par un ARC qui sera basé à Maradi pour la durée de l'étude. Il sera chargé de superviser les activités quotidiennes de logistique et de collecte des données des 3 sites de l'étude, conformément au protocole de l'étude et aux procédures opératoires standardisés. Il sera en communication permanente avec l'investigateur principal et le coordinateur médical et scientifique à Niamey, afin de les informer régulièrement de l'avancement de l'étude (par exemple le nombre d'admissions et de visites de suivi, le nombre de non présentations aux visites de suivi, l'état des stocks de matériel et médicaments, l'avancement de la saisie des données).
En cas de difficulté, il interrogera l'investigateur principal afin d'éviter les déviations par rapport au protocole, et il l'informera des écarts au protocole. L'état d'avancement de l'étude sera communiqué par écrit dans un rapport mensuel.

FORSANI sera responsable de la prise en charge nutritionnelle et médicale au CRENA des enfants non éligibles dans l'étude ainsi que les enfants développant des complications et nécessitant une hospitalisation au CRENI.

Les résultats de l'étude seront régulièrement communiqués à tous les partenaires. Un article scientifique sera préparé et présenté au comité de lecture d'une revue spécialisée, ainsi qu'à des colloques internationaux, afin de permettre une large diffusion à la communauté scientifique.

7. CONSIDERATIONS ADMINISTRATIVES ET LOGISTIQUES

Les personnes directement impliquées dans l'administration de l'étude sont les suivantes.

Chaque équipe de l'étude sera composée de 1 médecin, 2 infirmier CRENA, 2 assistants nutritionnels, 1 infirmier-préleveur, 1 assistant-pharmacien, 1 agent de santé communautaire, 1 chauffeur pour le site.

- L'investigateur principal, épidémiologiste

- L'ARC, médecin qualifié, avec expérience en recherche épidémiologique. Recruté par Epicentre, il sera chargé de la formation et la supervision du personnel de l'étude, il surveillera les activités quotidiennes de l'étude, conformément au protocole de l'étude.
- Le médecin, les 2 infirmiers et les 2 assistants nutritionnels seront responsables de l'inclusion et du suivi des enfants participant à l'étude sur leur site d'affectation. Les membres de l'équipe de l'étude seront recrutés localement pour la durée de l'étude.
- L'infirmier-préleveur, recruté localement, en charge de collecter les prélèvements biologiques sur les sites d'inclusion et lors des visites à domicile
- L'assistant pharmacien : un profil paramédical avec expérience en gestion de pharmacie recruté localement, chargé de distribuer les traitements de l'étude randomisée, en aveugle, et les médicaments de routine administrés pour les soins standards.
- L'agent de santé communautaire: un assistant recruté localement, chargé d'enregistrer les données résidentielles de tous les enfants inclus, de rechercher les enfants qui ne se sont pas présentés aux visites de suivi et de retourner aux domiciles des enfants inclus dans l'étude ancillaire.
- Deux opérateurs de saisie, sous la supervision du responsable de gestion des données, seront basés au bureau Epicentre, à Maradi, où ils assureront la saisie des données en continu et le nettoyage de la base de données.
- Le chauffeur : le chauffeur, recruté localement, connait la région de l'étude et accompagnera l'agent de santé communautaire
- Un assistant logisticien pour la supervision des installations logistiques de sites durant toute la période des inclusions
- Un assistant administratif pour tout le suivi des procédures RH et comptables durant l'étude.

L'équipe du laboratoire sera composée de 4 techniciens de laboratoire et un superviseur recrutés localement et chargés de préparer les milieux de culture, d'ensemencer les échantillons biologiques, d'identifier les bactéries pathogènes, de réaliser les antibiogrammes, de rendre les résultats biologiques aux médecins et de préparer les échantillons pour envoi au laboratoire extérieur. Cette équipe sera sous la responsabilité d'un responsable laboratoire expatrié pour la gestion de cette unité.

Tout le personnel recruté localement sera sous la supervision directe de l'ARC, mis à part le personnel du laboratoire qui sera sous la supervision directe du responsable du laboratoire. Les deux responsables collaboreront étroitement pour le bon déroulement des analyses nécessaires durant l'étude.

Les considérations logistiques pour la mise en œuvre de l'étude sont les suivantes:

- Espace suffisant, dans ou à côté du CRENA pour le dépistage, la consultation et la collecte des échantillons (1 salle d'attente, 2 salles de consultation équipées et une pièce confinée pour les prélèvements, par équipe de l'étude)
- Espace suffisant dans CRENA pour le stockage sûr (fermé à clé) des dossiers d'observation des enfants

- Espace suffisant dans CRENA pour le stockage sûr du matériel et des traitements de l'étude
- Fournitures médicales et nutritionnelles appropriées pour le traitement ambulatoire de la MAS
- Modalités de transfert définies entre l'étude et le programme nutritionnel FORSANI (CRENA et CRENI)
- 4 véhicules pour la recherche des patients et la livraison des prélèvements au laboratoire de Maradi
- 3 ordinateurs portables pour l'ARC et l'équipe de saisie des données
- Un réfrigérateur et un congélateur supplémentaire pour le stockage des souches bactériennes, des réactifs de laboratoire et des milieux de culture ainsi qu'un incubateur supplémentaire pour la culture, au sein du laboratoire de Maradi
- Indemnité appropriée à fournir aux mères à chaque visite

8. REFERENCES

1. World Health Organization (1999) Management of severe malnutrition: a manual for physicians and other senior health workers. Geneva: World Health Organization.

2. World Health Organization The WHO Child Growth Standards.

3. United Nations Children's Fund (2009) Tracking progress on child and maternal nutrition. New York: UNICEF.

4. Black RE, Allen LH, Bhutta ZA, Caulfield LE, de Onis M, et al. (2008) Maternal and child undernutrition: global and regional exposures and health consequences. Lancet 371: 243-260.

5. World Health Organization tWFP, the United Nations System Standing Comittee on Nutrition, and the United Nations Children's Fund (2007) Community-based management of severe acute malnutrition. Geneva: World Health Organization, the World Food Program, the United Nations System Standing Comittee on Nutrition, and the United Nations Children's Fund.

6. Collins S, Sadler K, Dent N, Khara T, Guerrero S, et al. (2006) Key issues in the success of community-based management of severe malnutrition. Food Nutr Bull 27: S49-82.

7. Médecins sans Frontières-France (2011) Implementation of emergency nutritional programs (internal document). Paris: Médecins sans Frontières-France.

8. Scrimshaw NS, SanGiovanni JP (1997) Synergism of nutrition, infection, and immunity: an overview. Am J Clin Nutr 66: 464S-477S.

9. Berkowitz FE (1983) Infections in children with severe protein-energy malnutrition. Ann Trop Paediatr 3: 79-83.

10. Lazzerini M, Tickell D (2011) Antibiotics in severely malnourished children: systematic review of efficacy, safety and pharmacokinetics. Bull World Health Organ 89: 594-607.

11. Bachou H, Tylleskar T, Kaddu-Mulindwa DH, Tumwine JK (2006) Bacteraemia among severely malnourished children infected and uninfected with the human immunodeficiency virus-1 in Kampala, Uganda. BMC Infect Dis 6: 160.

12. Berkley JA, Lowe BS, Mwangi I, Williams T, Bauni E, et al. (2005) Bacteremia among children admitted to a rural hospital in Kenya. N Engl J Med 352: 39-47.

13. Brent AJ, Ahmed I, Ndiritu M, Lewa P, Ngetsa C, et al. (2006) Incidence of clinically significant bacteraemia in children who present to hospital in Kenya: community-based observational study. Lancet 367: 482-488.

14. Hill PC, Onyeama CO, Ikumapayi UN, Secka O, Ameyaw S, et al. (2007) Bacteraemia in patients admitted to an urban hospital in West Africa. BMC Infect Dis 7: 2.

15. Hossain MI, Dodd NS, Ahmed T, Miah GM, Jamil KM, et al. (2009) Experience in managing severe malnutrition in a government tertiary treatment facility in Bangladesh. J Health Popul Nutr 27: 72-79.

16. Noorani N, Macharia WM, Oyatsi D, Revathi G (2005) Bacterial isolates in severely malnourished children at Kenyatta National Hospital, Nairobi. East Afr Med J 82: 343-348.

17. Howard D, Cordell R, McGowan JE, Jr., Packard RM, Scott RD, 2nd, et al. (2001) Measuring the economic costs of antimicrobial resistance in hospital settings: summary of the Centers for Disease Control and Prevention-Emory Workshop. Clin Infect Dis 33: 1573-1578.

18. Nys S, Okeke IN, Kariuki S, Dinant GJ, Driessen C, et al. (2004) Antibiotic resistance of faecal Escherichia coli from healthy volunteers from eight developing countries. J Antimicrob Chemother 54: 952-955.

19. Okeke IN, Laxminarayan R, Bhutta ZA, Duse AG, Jenkins P, et al. (2005) Antimicrobial resistance in developing countries. Part I: recent trends and current status. Lancet Infect Dis 5: 481-493.

20. Babirekere-Iriso E, Musoke P, Kekitiinwa A (2006) Bacteraemia in severely malnourished children in an HIV-endemic setting. Ann Trop Paediatr 26: 319-328.

21. Bejon P, Mwangi I, Ngetsa C, Mwarumba S, Berkley JA, et al. (2005) Invasive Gram-negative bacilli are frequently resistant to standard antibiotics for children admitted to hospital in Kilifi, Kenya. J Antimicrob Chemother 56: 232-235.

22. Meremikwu MM, Nwachukwu CE, Asuquo AE, Okebe JU, Utsalo SJ (2005) Bacterial isolates from blood cultures of children with suspected septicaemia in Calabar, Nigeria. BMC Infect Dis 5: 110.

23. Rabasa AI, Shattima D (2002) Urinary tract infection in severely malnourished children at the University of Maiduguri Teaching Hospital. J Trop Pediatr 48: 359-361.

24. De Rekeneire N (2008) Etude de la prevalence des infections chez les enfants severement malnutris. Paris: Epicentre.

25. Trehan I, Amthor RE, Maleta K, Manary MJ (2010) Evaluation of the routine use of amoxicillin as part of the home-based treatment of severe acute malnutrition. Trop Med Int Health.

26. Manary M, Maleta K, Trehan I (2012) Randomized, double-blind, placebo-controlled trial evaluating the need for routine antibiotics as part of the outpatient management of severe acute malnutrition. Report to funder, Food and Nutrition Technical Assistance II Project (FANTA-2): Washington University in St-Louis

27. Ministere de la sante publique (2012) Protocole national de la prise en change integree de la malnutrition aiguë. Niamey: Ministere de la sante publique.

Annexes

Lettre d'information

Formulaire de consentement éclairé

Procédures de councelling VIH

Formulaire de consentement éclairé pour le dépistage VIH

Lettre d'information de la sous-étude ancillaire

Formulaire de consentement éclairé de la sous-étude ancillaire

Lettre d'information

L'effet de l'utilisation systématique d'un antibiotique dans le traitement en ambulatoire des enfants sévèrement malnutris sans complication

But de l'étude

Cette étude a pour but de montrer l'équivalence des deux moyens de traiter certaines infections chez les enfants sévèrement malnutris sans complication admis dans les programmes nutritionnels. Les résultats vont permettre de mieux prendre en charge les enfants malnutris à l'avenir dans les programmes nutritionnels.

Procédures de l'étude

Votre enfant va recevoir soit un antibiotique à l'entrée dans le programme nutritionnel qui est toujours le même que dans les autres centres, celui qui est donné habituellement (l'amoxicilline à prendre pendant 7 jours), soit un placebo (préparation inactive) pendant 7 jours et un antibiotique uniquement au moment où il en a besoin, selon la prescription du médecin employé par Epicentre. Votre enfant sera examiné par un médecin chaque semaine jusqu'à la sortie du programme nutritionnelle, puis une fois par mois pendant trois mois. A chaque consultation, le médecin pourra adapter le traitement, pour qu'il soit le meilleur pour votre enfant. Votre enfant recevra les mêmes soins que fournis par le programme nutritionnel habituel, mais il sera vu par un médecin spécifique travaillant dans l'étude. Un infirmier collectera le sang, les selles, l'urine et un écouvillon nasal à votre enfant au moment de l'inclusion pour procéder à des analyses au laboratoire pour aider à mieux le traiter. Ces prélèvements pourront être renouvelés si votre enfant développe de complications médicales durant son suivi dans l'étude. Si vous ne vous présentez pas aux visites de suivi programmées qui vous seront communiqués, une équipe de l''étude pourra se rendre à votre domicile afin de voir si tout va bien pour l'enfant.

Confidentialité

L'interrogatoire est confidentiel. Le nom de votre enfant ne sera pas utilisé lors de la saisie des données dans l'ordinateur, et personne en dehors du médecin et des responsables de l'étude ne verra les informations recueillies.

Coût et paiement

Tous les traitements, les soins et les analyses de votre enfant seront gratuits. On vous demandera de revenir toutes les semaines jusqu'à sa guérison, puis une fois par mois pendant trois mois de suivi. Vous vous engagez donc à faire votre possible pour respecter tous les rendez-vous pour le suivi. Nous savons que cela peut causer des désagréments, donc nous vous donnerons trois morceaux du savon à chaque visite prévu au CRENA et trois pagnes à la fin du suivi comme dédommagement. Le dédommagement ne sera pas donné lors des visites à domicile.

Libre choix de participation

Vous êtes libre d'accepter ou de refuser que votre enfant participe à l'étude, sans aucun préjudice ni reproche vis-à-vis de votre enfant ou de vous-même, et sans avoir à donner de raison. Si vous ne voulez pas participer à l'étude, votre enfant sera pris en charge comme tous les autres enfants dans le programme nutritionnel habituel. Vous pouvez maintenant, et pendant toute l'étude, poser toutes les questions à l'infirmier et au médecin si vous n'avez pas compris certaines choses ou si vous voulez plus d'information : ils sont là pour vous aider.

Merci beaucoup pour votre aide ! En cas de problème ou de question, merci de contacter :

Point focale du CRENA:	___________________	_______________
Médecin responsable de l'étude:	Dr Fatou Berthé	98967251

Formulaire de consentement éclairé
L'effet de l'utilisation systématique d'un antibiotique dans le traitement en ambulatoire des enfants sévèrement malnutris sans complication

Nom de l'infirmier recueillant le consentement :……………………………………………………………………………………..

Je soussigné (e)

Prénom …………………………………………………………………Nom…………………………………………………………………..

mère ☐ père ☐ représentant légal ☐

de l'enfant :

Prénom …………………………………………………………………Nom…………………………………………………………………..

Déclare:
Avoir lu (ou avoir reçu par un témoin qui me l'a lue) la note d'information qui concerne l'enquête sur l'étude Epicentre ***l'effet de l'utilisation systématique d'un antibiotique dans le traitement en ambulatoire des enfants sévèrement malnutris sans complication.***

J'ai compris les objectifs de l'étude et son déroulement. J'ai obtenu des réponses claires à toutes mes questions.

J'ai aussi compris que je peux refuser de participer à l'étude sans avoir à fournir de raison et sans aucun préjudice ni reproche vis-à-vis de mon enfant et de moi-même. Je suis d'accord pour participer à l'étude sous les conditions présentées dans la note d'information.

Signature ou empreinte digitale* :

Lieu ………………………………………….. Date ……../………/…….

*Si le parent ou représentant légal ne peut lire ou écrire, un témoin impartial doit être présent pendant la discussion pour le consentement éclairé. Une fois que la note de consentement éclairé a été lue et expliquée, et une fois que le parent/représentant légal a oralement consenti à la participation de l'enfant à l'étude, et signé ou apposé son empreinte digitale, le témoin doit signer et dater le formulaire de consentement éclairé. En signant, le témoin confirme que l'information a été correctement transmise au parent/représentant légal, et apparemment comprise par le parent/représentant légal, et que le consentement éclairé a été donné en toute liberté.

Témoin de consentement éclairé :

Prénom …………………………………………………………………Nom……………………………………………………………………

Signature:

|__|-|__|__|__|
Numero d'identification de l'enfant

Procédures de counseling VIH

Le counseling sera toujours réalisé par un personnel de santé formé au préalable. Cette personne instaurera une relation de confiance avec la mère de l'enfant, les explications données seront simples, précises, claires, adaptées au niveau de compréhension et à la langue de la mère. La personne délivrant le counseling n'aura en aucun cas une attitude de jugement.

Le counseling sera réalisé en trois étapes :

- Counseling pré-test
- Réalisation du prélèvement
- Counseling post-test

Après information, la décision de réaliser le test de dépistage du VIH sur l'enfant sera prise par la mère ou le responsable légal de l'enfant : c'est un acte volontaire et son choix sera respecté. Le test sera proposé à l'enfant malnutri uniquement.

Si on estime que la compréhension est mauvaise, ou qu'un résultat positif peut avoir des conséquences dramatiques pour la mère et l'enfant, on pourra reporter le test et proposer un counseling ultérieur, éventuellement en présence d'un autre proche de la mère.

Si la mère ou le responsable légal de l'enfant refuse le test pour l'enfant, une information lui sera donnée sur l'infection VIH/sida, avec conseils de prévention pour la réduction des risques, et orientation vers d'autres services de santé si nécessaire. Le refus du dépistage VIH ne modifiera en rien la qualité de la prise en charge médico-nutritionnelle de l'enfant. Le responsable de l'enfant pourra à tout moment revenir si elle change d'avis et souhaite réaliser le dépistage.

Counseling pré-test

L'information donnée avant le consentement éclairé sera toujours faite de façon individuelle, en privé par un personnel de santé formé au préalable. Cette information sera donnée à la mère de l'enfant ou à son responsable légal. Il est primordial d'instaurer une relation de confiance entre les personnes. L'information donnée sera simple, claire et précise, adaptée au niveau de compréhension de la personne participante.

Le personnel de santé réalisant le counseling donnera toujours les informations suivantes :

- Le motif de la proposition de la réalisation du dépistage, à savoir la malnutrition et les complications médicales associées.
- Le bénéfice clinique de connaître le statut VIH de l'enfant, qui pourra bénéficier précocement d'une prise en charge et de traitements adaptés.
- La prise en charge, conseils de prévention et services proposés en cas de séropositivité pour le VIH, incluant les traitements antirétroviraux.

- Les risques potentiels en cas de séropositivité, tels que la discrimination et les conséquences familiales.
- Les résultats seront toujours rendus de façon confidentielle, et le secret médical et la confidentialité seront respectés dans tous les cas.
- La mère ou le responsable légal de l'enfant peut à tout moment refuser le test, il ne sera réalisé que si elle donne son accord.
- Le refus du test ne va pas empêcher l'enfant d'avoir accès à la même prise en charge nutritionnelle et médicale que les autres enfants.
- La mère pourra si elle le souhaite garder secret le résultat du dépistage, en particulier vis à vis de sa famille.
- C'est l'opportunité pour la mère de poser toutes les questions sur le VIH/SIDA (transmission, prévention, prise en charge.)

L'information donnée à la mère ou au responsable légal de l'enfant portera également sur les mesures préventives en cas de grossesse :

- Le risque de transmission du VIH à leur enfant
- Les mesures pouvant être prises pour réduire la transmission mère-enfant, y compris grâce au traitement prophylactique par antirétroviraux et conseils sur l'allaitement maternel
- Les bénéfices pour l'enfant d'un diagnostic précoce.

Après les explications données sur la maladie, sa prise en charge, les traitements proposés, l'observance et le suivi régulier nécessaires, les difficultés psycho-sociales, la réalisation du dépistage sera proposée. En cas d'accord de la mère ou du responsable légal, un consentement sera signé avant la réalisation du prélèvement. Les enfants étant mineurs, le consentement sera signé par la mère de l'enfant, ou le responsable légal de l'enfant.

Réalisation du test de dépistage

Le test de dépistage sera réalisé pour l'enfant après acceptation et signature du consentement éclairé par la mère ou le responsable légal de l'enfant.
Les tests rapides seront réalisés sur place au moment du prélèvement. En cas de discordance entre les 2 tests rapides, un nouvel échantillon sera prélevé au bout du doigt pour analyse complémentaire dans un laboratoire extérieur (technique ELISA). En cas de positivité ou de discordances des tests pour un enfant de moins de 18 mois, un nouvel échantillon sera prélevé au bout du doigt pour analyse complémentaire dans un laboratoire extérieur (technique PCR).

Le délai nécessaire pour obtenir les résultats de confirmation sera donné à la mère, et un rendez-vous sera fixé pour le rendu des résultats (autant que possible lors d'une visite programmée de l'étude). La mère sera informée de la possibilité de revenir si elle a des questions ou si elle souhaite obtenir d'autres informations.

Post test counseling

Toutes les mères ou responsables légaux qui acceptent de réaliser un dépistage pour leur enfant doivent recevoir une information complète lorsque les résultats leur sont rendus, adaptée au résultat positif ou négatif.

En cas de négativité du test de dépistage (1er test négatif):
- Explications sur les méthodes de prévention de la transmission de l'infection par le VIH.
- Informer la mère ou le responsable légal qu'il est possible de réaliser un dépistage du VIH pour eux-mêmes, leur partenaire et les autres enfants de la famille à l'Hôpital Régional de Maradi, où il est également proposé les traitements et les mesures préventives comme la Prévention de la Transmission Mère-Enfant.
- Réponses à ses questions concernant son enfant et elle-même.

En cas de positivité du test (2 tests positifs et enfant > 18 mois):
- Informer de façon simple et claire sur la positivité du résultat, et s'assurer de la bonne compréhension de ce résultat.
- Avoir une attitude d'écoute, lui laisser exprimer son ressenti, ses difficultés, lui laisser le temps nécessaire. Aider la mère à gérer ses émotions au moment de l'annonce du résultat.
- Réexpliquer la différence entre la séropositivité par le VIH et le sida, ainsi que l'évolution de la maladie, en insistant sur le fait qu'elle peut être longtemps asymptomatique, et que les traitements par antirétroviraux existent et permettent parfois de stabiliser la situation.
- Décrire toute la prise en charge possible (en particulier celle proposée à l'Hôpital Régional de Maradi) et le suivi médical
- Informer la mère ou le responsable légal qu'il est possible de réaliser un dépistage du VIH pour eux-mêmes, leur partenaire et les autres enfants de la famille à l'Hôpital Régional de Maradi, où il est également proposé les traitements et les mesures préventives comme la Prévention de la Transmission Mère-Enfant.
- Demander à la mère avec qui elle souhaite partager ce résultat, essayer de trouver un proche dans l'entourage qui puisse partager l'annonce du résultat et la soutenir (parent, autre membre de la famille, représentant religieux, ami). La préparer à cette annonce, en lui expliquant comment elle peut la faire. Evoquer les difficultés familiales possibles, les abandons par les maris, le risque de violences faites aux femmes lors de l'annonce de la séropositivité dans la famille. Savoir la conseiller de garder ce résultat secret si on estime que la situation sera plus difficile pour elle si elle en parle dans son entourage.
- Réexpliquer les mécanismes de transmission du VIH et les moyens de prévention, en particulier l'utilisation des préservatifs.
- Réexpliquer l'importance des mesures de santé préventives associées comme le maintien d'un bon statut nutritionnel, l'utilisation du cotrimoxazole pour la prévention des maladies opportunistes, la prévention du paludisme.

Cas de besoins de tests complémentaires (ELISA ou PCR)
- Informer de façon simple et claire que vous ne pouvez pas vous prononcer sur le résultat car des analyses complémentaires sont nécessaires pour connaitre le véritable statut VIH de l'enfant.

Expliquer la façon dont les prélèvements complémentaires vont être réalisés, et s'assurer de la bonne compréhension.

- Informer que les analyses complémentaires vont être réalisés à Maradi ou à Niamey et que les prélèvements resteront anonymes (le nom de l'enfant ne figurera sur aucun document ou prélèvement)
- Informer la mère ou le responsable légal du délai de réception des résultats définitifs sur le statut VIH de leur enfant ; ces résultats leur seront rendus au cours du visite planifiée.
- Informer la mère ou le responsable légal qu'il est possible de réaliser un dépistage du VIH pour eux-mêmes, leur partenaire et les autres enfants de la famille à l'Hôpital Régional de Maradi, où il est également proposé les traitements et les mesures préventives comme la Prévention de la Transmission Mère-Enfant.
- Réexpliquer les mécanismes de transmission du VIH et les moyens de prévention, en particulier l'utilisation des préservatifs.

Formulaire de consentement éclairé pour le dépistage VIH

Je soussigné (e) nom……………………………..…………………prénom………………………………………………………………

mère ☐ père ☐ représentant légal ☐ en qualité de ……………………..…………………………..

de l'enfant : nom…………………………………………..………prénom…………………………………………………………..…

déclare avoir reçu des explications concernant l'infection par le VIH/Sida,
déclare avoir été informé(e) de la possibilité d'accepter ou de refuser le dépistage pour mon enfant,
déclare avoir reçu des explications sur la prise en charge médicale et le suivi de mon enfant si je le souhaite.
déclare avoir été informé(e) du fait que le résultat du test de dépistage demeurera confidentiel.
L'information confidentielle concernant le diagnostic d'infection par le VIH ne pourra être fournie qu'aux personnes qui doivent connaître l'état de santé de mon enfant afin d'offrir à mon enfant des soins et des services médicaux appropriés.

J'ai obtenu les réponses à mes questions concernant le test de dépistage du VIH, et j'accepte de faire faire à mon enfant.

Signature ou empreinte digitale* Lieu Date

*Si le parent ou représentant légal ne peut lire ou écrire, un témoin impartial doit être présent pendant la discussion pour le consentement éclairé. Une fois que la note de consentement éclairé a été lue et expliquée, et une fois que le parent/représentant légal a oralement consenti à faire le test de dépistage de VIH, et signé ou apposé son empreinte digitale, le témoin doit signer et dater le formulaire de consentement éclairé. En signant, le témoin confirme que l'information a été correctement transmise au parent/représentant légal, et apparemment comprise par le parent/représentant légal, et que le consentement éclairé a été donné en toute liberté.

<u>Témoin du consentement éclairé :</u>
Prénom, NOM : ……………………………………………………….
Signature

<u>Personne ayant recueillie le consentement</u> :
Prénom, NOM …….
Je certifie avoir offert au participant la consultation préalable au test de dépistage du SIDA. J'ai répondu aux questions de la personne mentionnée ci-dessus en ce qui concerne le test.
Je m'engage à respecter scrupuleusement le protocole avec les exigences de confidentialité et de respect des droits de la personne et de sa liberté qu'il implique.

Signature Lieu Date

|__|-|__|__|__|
Numero d'identification de l'enfant

Lettre d'information : Etude ancillaire

Prévalence, émergence et transmission de résistances aux antibiotiques chez des enfants sévèrement malnutris sans complication et leur fratrie

But de l'étude

Votre enfant a été inclus dans une étude qui a pour but de montrer l'équivalence des deux moyens de traiter certaines infections chez les enfants sévèrement malnutris sans complication admis dans les programmes nutritionnels. Une sous-étude est menée sur la résistance aux antibiotiques dans un sous-groupe d'enfants de l'étude et leurs frères et sœurs âgés de 6 à 59 mois. L'objectif est de décrire la présence éventuelle de bactéries résistantes aux antibiotiques dans les selles des enfants de l'étude au moment de l'inclusion, et après 7 et 28 jours et également leur éventuelle transmission aux frères/ sœurs.

Procédures de l'étude

Parmi les enfants inclus dans l'étude générale, un enfant par jour sera inclus au hasard dans la sous-étude, trois fois par semaine. Les selles de votre enfant seront prélevés le 1er jour (jour de l'inclusion) puis au 7ème et au 28ème jour lors des visites régulières au CRENA. Un maxiumum de 4 enfants de la fratrie, ayant entre 6 et 59 mois seront également inclus dans la sous-étude. S'il y a plus de 4 enfants éligibles dans la fratrie, ils seront sélectionnés au hasard. Leurs selles seront également prélevées le 1er jour, puis le 7ème jour et enfin le 28ème jour. A la fin de la visite d'inclusion, du 7ème jour et du 28^{ème}jour au CRENA, vous et votre enfant seront raccompagnés à votre domicile en véhicule par une équipe de l'étude qui profitera de ce moment pour prélever les frères et sœurs s'ils ne vous accompagnent pas lors des visites au CRENA. Il pourra éventuellement vous être demandé de prélever vous-même les selles de vos enfants si l'équipe de l'étude n'a pas pu les prélever ce jour dans un temps raisonnable. Pour cela, nous vous expliquerons comment faire et vous fournirons tout le matériel nécessaire. Les selles de vos enfants pourront être conservées et utilisées pour des analyses complémentaires.

Confidentialité

L'interrogatoire est confidentiel. Les noms des enfants ne seront pas utilisés lors de la saisie des données sur ordinateur, et personne en dehors de l'étude ne verra les informations recueillies.

Coût et paiement

Toutes les analyses biologiques réalisées sur les prélèvements de vos enfants seront gratuites. On vous demande de revenir au 7ème et au 28ème jour de suivi avec l'enfant inclus dans l'étude, et si possible, avec les frères et les sœurs inclus dans la sous-étude. Vous vous engagez donc à faire votre possible pour respecter tous les rendez-vous pour le suivi. Vous ne recevrez aucun paiement pour participer à la sous-étude mais vous bénéficierez d'un savon à chaque visite au CRENA comme il est prévu dans le cadre de l'étude générale.

Libre choix de participation

Vous êtes libre d'accepter ou de refuser que vos enfants participent à la sous-étude, sans aucun préjudice ni reproche vis-à-vis de votre enfant ou de vous-même, et sans avoir à donner de raison. Si vous ne voulez pas participer à la sous-étude, votre enfant sera pris en charge normalement dans l'étude générale. Vous pouvez maintenant, et pendant toute l'étude, poser toutes les questions à l'infirmier et au médecin si vous n'avez pas compris certaines choses ou si vous voulez plus d'informations : ils sont là pour vous aider.

Merci beaucoup pour votre aide ! En cas de problème ou de question, merci de contacter :

Médecin du CRENA d'inclusion : ………………………………………………….
Médecin responsable de l'étude : Dr Fatou Berthé 98 9672 51

Formulaire de consentement éclairé : Etude ancillaire

Prévalence, émergence et transmission de résistances aux antibiotiques chez des enfants sévèrement malnutris sans complication et leur fratrie

Nom de l'infirmière recueillant le consentement ………………………………………………………………………….

Je soussigné (e)

Prénom ………………………………………………………………….Nom………………………………………………………………….

mère ☐ père ☐ représentant légal ☐

de l'enfant :

Prénom ……………………………………………………………Nom…………………………………………………………………..

Déclare:
Avoir lu ou avoir reçu par un témoin qui me l'a lue la note d'information qui concerne la sous-étude de résistance aux antibiotiques dans l'étude Epicentre ***d'équivalence de deux modalités d'antibiothérapie chez les enfants sévèrement malnutris traités en ambulatoire***.

J'ai compris les objectifs de la sous-étude et son déroulement. J'ai obtenu des réponses claires à toutes mes questions.

J'ai aussi compris que je peux refuser de participer à la sous-étude sans avoir à fournir de raison et sans aucun préjudice ni reproche vis-à-vis de mon enfant et de moi-même. Je suis d'accord pour participer à la sous-étude sous les conditions présentées dans la note d'information.

Signature ou empreinte digitale* :

Lieu …………………………………………….. Date ……../………/…….

*Si le parent ou représentant légal ne peut lire ou écrire, un témoin impartial doit être présent pendant la discussion pour le consentement éclairé. Une fois que la note de consentement éclairé a été lue et expliquée, et une fois que le parent/représentant légal a oralement consenti à la participation de l'enfant à l'étude, et signé ou apposé son empreinte digitale, le témoin doit signer et dater le formulaire de consentement éclairé. En signant, le témoin confirme que l'information a été correctement transmise au parent/représentant légal, et apparemment comprise par le parent/représentant légal, et que le consentement éclairé a été donné en toute liberté.

Témoin de consentement éclairé :

Prénom ………………………………………………………………….Nom………………………………………………………………….

Signature :

|__|-|__|__|__|
Numero d'identification de l'enfant
